# Nitridated Iron-Based (Nano)Materials for Environmental Remediation: Synthesis, Characterization, and Performance

**DOI:** 10.1021/acs.est.5c10601

**Published:** 2025-11-14

**Authors:** Li Gong, Jingting Chen, Feng He, Miroslav Brumovský, Jan Filip, Paul G. Tratnyek

**Affiliations:** † Zhejiang Key Laboratory of Low-carbon Control Technology for Industrial Pollution, College of Environment, 12624 Zhejiang University of Technology, Hangzhou 310014, China; ‡ School of Environment and Ecology, Jiangnan University, Wuxi 214122, China; § Regional Centre of Advanced Technologies and Materials, Czech Advanced Technology and Research Institute, Palacký University Olomouc, Olomouc CZ-78371, Czech Republic; ∥ OHSU-PSU School of Public Health, 6684 Oregon Health & Science University, Portland, Oregon 97239, United States

**Keywords:** thermochemical nitridation, mechanochemical nitridation, zero-valent iron, iron nitrides, Fe–N coordination structures

## Abstract

Iron-based materials, particularly zerovalent iron (ZVI), are widely used in environmental remediation. Still, contaminant removal can be limited by surface passivation and nontarget side reactions that compromise treatment efficiency and sustainability. To address these limitations, many modifications of ZVI have been proposed, including, most recently, nitridation (i.e., incorporation of N on the surface or within the bulk of ZVI into iron lattice). This review examines methods of nitridation (including thermochemical and mechanochemical nitridation), their effects on the morphology/physicochemical properties of iron-based materials, and the performance of the resulting materials for reduction of contaminants. Nitridation leads to the formation of iron nitrides and/or Fe–N coordination structures, enhancing dechlorination performance through different mechanisms. Iron nitrides improve electron transfer efficiency, suppress the hydrogen evolution reaction, and accelerate reductive dechlorination of a broad range of contaminants. In contrast, Fe–N coordination structures facilitate proton transfer, leading to improved dechlorination, but with different kinetic characteristics. Additionally, nitridation extends the reactive lifespan of ZVI by mitigating passivation, with iron nitrides offering direct corrosion resistance. This review highlights the potential of nitridated iron-based materials for efficient, selective, and durable remediation applications, and identifies areas for further research required to enable their widespread adoption in full-scale environmental restoration.

## Graphical abstract

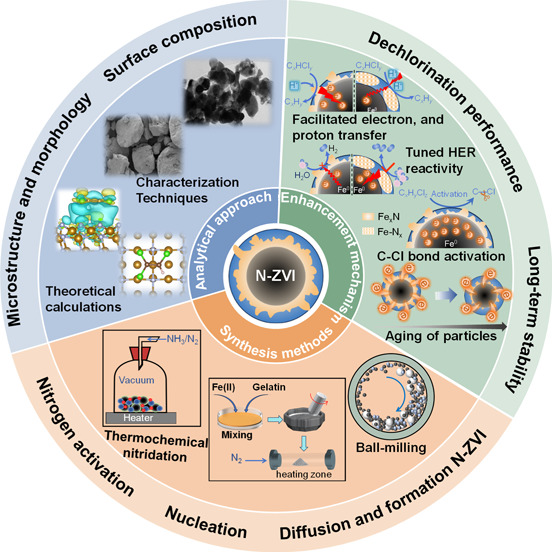

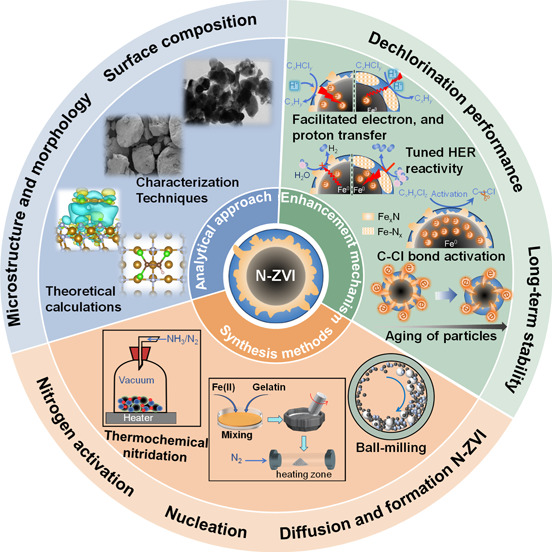

## 1 Introduction

Zero-valent iron-based (nano)­particles ((n)­ZVI) are used in a wide range of applications, including environmental remediation, chemical synthesis, and energy storage.^1–11^ However, their overall performance in these applications can be limited by several characteristics, including low-to-high (but nonspecific) reactivity, susceptibility to corrosion (generally an unproductive side reaction), and rapid agglomeration (particularly in the case of nZVI). To overcome these challenges, many surface and/or bulk chemical modification techniques have been investigated in the laboratory, and some have proven to be effective under field-scale conditions.^12,13^ Among these enhancements to (n)­ZVI-based remediation technologies, nitridation has recently emerged as one of the most effective and promising.

Nitridation consists of incorporating N on the surface or within the bulk of metals, which can be accomplished by various methods. For instance, the diffusion-controlled nitridation at elevated temperatures, also referred to as nitriding,^14^ is well-known in metallurgy as a surface hardening process that enhances the wear resistance, fatigue strength, and corrosion resistance of metals.^15–17^ This process is commonly used in the automotive,^18^ aerospace, tooling,^19^ and manufacturing industries to improve the durability and performance of metal components. Nitriding involves the incorporation of nitrogen into the lattice of metal materials, leading to the formation of crystalline iron nitrides (Fe_*x*_N), which have unique electronic properties, high conductivity, and chemical stability.^20,21^ These properties have further driven the application of nitridated iron-based materials across multiple disciplines, ranging from magnetic recording heads,^20^ through catalysis,^21^ to biomedicine.^22^ Notably, iron nitrides serve not only as reductants, but also are known to catalyze redox reactions due to their noble-metal-like electronic behavior (where metal lattice expansion-induced d-band contraction increases the density of states near the Fermi level).^21,23,24^ Recently developed methods of preparing nitridated ZVI (N-ZVI) with their targeted application in environmental technologies have produced materials that preferentially adsorb contaminants, thereby further enhancing the performance of ZVI in environmental remediation.^25–29^ And, unlike the passivating oxide layers that hinder the outward electron transfer from the Fe^0^ core in conventional ZVI,^30–34^ the Fe_*x*_N formed on the surface of N-ZVI (or in the entire particle volume) is relatively conductive, thereby favoring outward electron transfer to sorbed contaminants.^26,29^

Methods of nitridating iron-based precursors for the preparation of N-ZVI materials have been developed mainly through adopting established surface engineering approaches from metallurgy and related industries, where solid-state,^35^ salt-bath,^36^ gas,^37^ and plasma nitriding^38^ are utilized to incorporate N into iron-based materials by different nitridation agents. During the nitridation process, nitrogen can incorporate into iron lattice while forming distinct crystalline phases (e.g., body-centered cubic (BCC) α″-Fe_16_N_2_, face-centered cubic (FCC) γ′-Fe_4_N, and hexagonal close-packed (HCP) ε-Fe_3_N, Figure 1a), with distinct electronic and mechanical properties.^20,25–27^ The crystal structure of γ′-Fe_4_N adopts a FCC configuration with nitrogen atom as N^3–^ occupying 1/4 of octahedral sites and the iron atoms (existing in two inequivalent sites) formally in slightly positive state on average +0.75. In contrast, the ε-Fe_3_N adopts an HCP structure, with N^3–^ occupying 1/3 of the octahedral sites and the iron atoms exhibiting a formal oxidation state of +1. However, the Fe–N interaction is more covalent than ionic because the effective positive charges on Fe atoms are smaller.^24^ The crystal structure, composition, particle size, and morphology of Fe_*x*_N can be finely tuned by controlling nitridation parameters, including temperature, pressure, reaction time, and nitrogen source selection in combination with the selection of solid precursor.^14,20,26,29^ This enables the fine-tuning of Fe_*x*_N materials to achieve specific catalytic and redox characteristics suitable for particular environmental applications.

**Figure 1:**
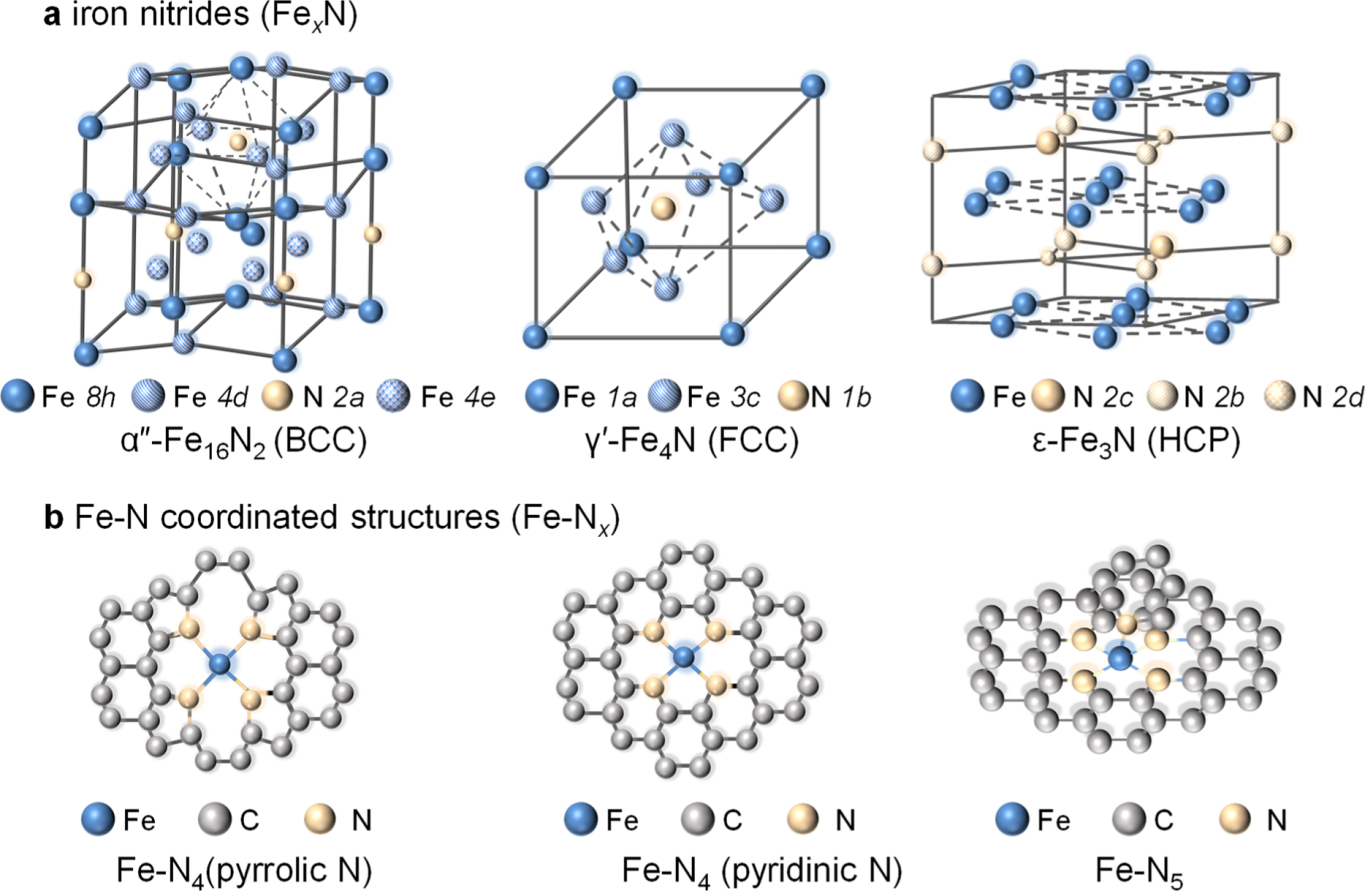
Structural configurations of (a) iron nitride crystalline phases,^20^ (b) Fe–N coordination complexes with distinct coordination geometries.^39^

In addition to crystalline iron nitrides, nitridation of iron precursors by N-rich organic compounds at mild temperatures can also lead to the formation of carbon structures with Fe–N coordination sites (denoted as Fe–N_*x*_, where *x* represents the number of nitrogen atoms coordinated to iron within pyridinic or pyrrolic types of sites), as illustrated in Figure 1b.^40^ These Fe–N_*x*_ coordinated species have been extensively studied as active sites in natural enzymes and Fe single-atom catalysts (commonly designated as Fe–N–C^41–44^ ) for the catalytic conversion of organic compounds/waste into value-added chemicals. Furthermore, the Fe–N_*x*_ coordinated structure in Fe–N–C has been shown to catalyze the degradation of some organic pollutants via reduction or oxidation reactions.^45–47^ Taking advantage of these characteristics, it has been confirmed that combining Fe–N_*x*_ coordination structures with ZVI can result in catalytic reductive dechlorination of contaminants.^29,48–50^

Comparative studies over the past five years have shown that nitridation of ZVI has similar benefits as sulfidation (currently, the most extensively studied and promising ZVI modification, with sulfidated ZVI referred to as S-ZVI^51–58^ ), without some of the drawbacks (like the health/safety issues from working with sulfides). Nitridation also has some unique benefits over sulfidation, in particular higher reactivity with prevalent contaminants such as tetrachloroethylene (PCE), *cis*-1,2-dichloroethylene (*cis*-DCE),^25^ and chloroform (CF)^59^ and broader operational pH range. Therefore, it is informative to summarize recent advances in the nitridation of iron-based materials for pollutant transformation. This review explores nitridation methods, mechanisms, material properties, and pollutant removal performance, aiming to illuminate the potential of N-ZVI, which has been relatively less explored compared to S-ZVI.

## 2 Fundamentals of Nitridation of Zero-Valent Iron

### 2.1 Nitridation Methods

Unlike sulfidation, which is primarily achieved through liquid-phase or mechanochemical methods,^52,55,60^ nitridation predominantly employs thermochemical^48–50,61^ or mechanochemical^26,29^ nitridation methods due to nitrogen’s low solubility and high activation barrier. The following sections will discuss these two main approaches in detail, as they have been applied in recent studies to synthesize N-ZVI for remediation applications. In principle, other nitridation techniques used in metallurgy, such as cold plasma treatment in NH_3_ or N_2_ atmospheres, could also be adapted for this purpose.

#### 2.1.1 Thermochemical Nitridation

Thermochemical nitridation processes are widely used for modifying iron-based materials with iron nitrides. Typically, thermochemical nitridation involves introducing nitrogen atoms into the metal surface through diffusion by exposure to nitrogen-rich gases (even evolved from decomposition of N-bearing organic compounds) at elevated temperatures (typically 350–600 °C) in a controlled environment (Table S1). Nitrogen reacts with iron to form iron nitrides (e.g., γ′-Fe_4_N, ε-Fe_3_N). Based on the nitrogen precursor, thermochemical nitridation procedures reported in available studies dealing with N-ZVI can be categorized into gas–solid^26^ and solid–solid nitridation^29^ processes.

In gas–solid thermochemical nitridation, ZVI powder is exposed to nitrogen-rich gases such as NH_3_ (or NH_3_ mixtures, e.g., with N_2_) within controlled closed reactors or tube furnaces (Figure 2a and Table S1). At elevated temperatures, the breakdown of N=N and N–H bonds of the gases on the surface of iron particles occurs, releasing atomic nitrogen, followed by nitrogen diffusion into the iron lattice, forming iron nitride phases such as γ′-Fe_4_N and ε-Fe_2–3_N.^26,28^ The specific phases formed depend on parameters such as reaction temperature,^26^ gas pressure,^62^ and nitriding gas composition.^62^ For instance, γ′-Fe_4_N can be formed from commercial nZVI particles (dry powder) at 430–500 °C under 0.5 bar after 3 h of nitridation in a fluid laboratory furnace using NH_3_/N_2_ gas mixture containing 30–50% NH_3_, whereas ε-Fe_2–3_N is predominantly formed at 300 °C using NH_3_/N_2_ 2:1 gas mixture after 5.5 h of nitridation.^26^ Although elevated temperatures enhance nitrogen diffusion, excessively high temperatures can lead to undesired effects such as the diffusion of nitrogen out from the lattice or particle sintering, as observed at temperatures above 1000 °C.^17,63,64^ In the kinetically controlled phase, the composition of formed Fe_*x*_N also depends on the reaction time and flow rate of nitriding gas.^62,65,66^ Additionally, the material properties of the iron precursors, such as particle size, shape, and porosity, strongly influence the nitridation process. For example, needle-like γ′-Fe_4_N and α-Fe were observed when iron sheets were nitridated for 2 h under NH_3_/H_2_ gas mixtures at 840 °C,^67^ whereas spherical γ′-Fe_4_N particles were produced using iron powder under similar conditions.^68^

**Figure 2:**
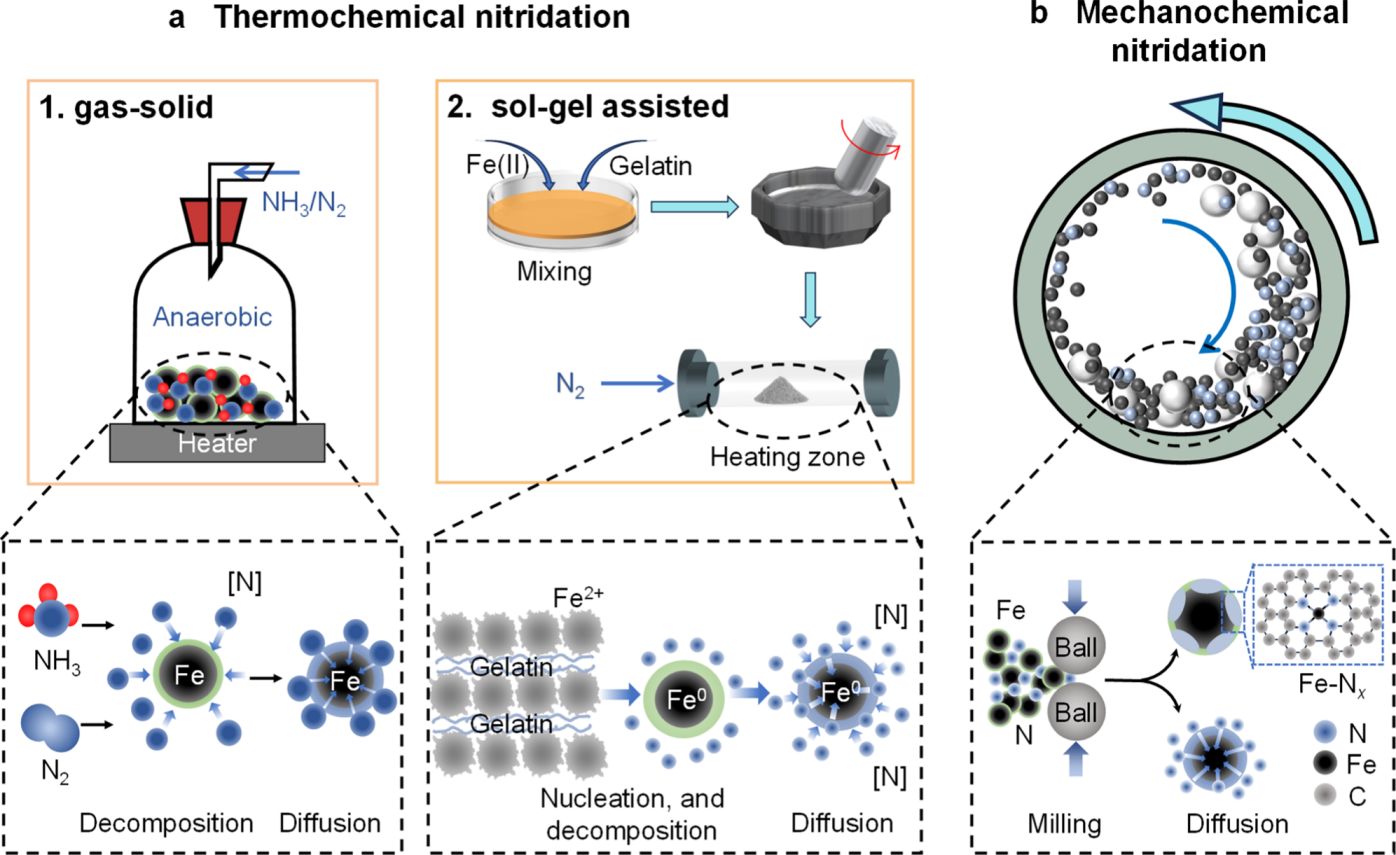
Two main methods for synthesizing N-ZVIs for water treatment: (a) the thermochemical nitridation method (including gas–solid nitridation and solid–solid nitridation methods), and (b) the mechanochemical nitridation method.

The solid–solid thermochemical nitridation method employs a preparation of iron/iron-oxide (nano)­particles embedded in a solid N-bearing organic compound, followed by pyrolysis to achieve nitrogen diffusion into zerovalent iron.^29,69,70^ As an example, a colloidal sol is prepared using ferric precursors (C_4_H_6_FeO_4_) and gelatin as the nitrogen source (Figure 2a and Table S1).^29^ The process begins with the gelation of the sol, which is induced through pH adjustments, resulting a uniform distribution of iron ions within the matrix. After drying, the formed xerogel is pyrolyzed under an inert or reducing atmosphere, facilitating the reduction of Fe^3+/^Fe^2+^ to Fe^0^ while simultaneously incorporating nitrogen (from thermally decomposed gelatin) into the iron lattice to form iron nitrides.^29^ The nitridation pathway with regard to the formation of atomic N in the solid–solid method follows a mechanism different from that of gas–solid nitridation, as nitrogen is supplied from the decomposition of gelatin rather than from a gaseous precursor.

#### 2.1.2 Mechanochemical Nitridation

In mechanochemical synthesis, mechanical energy is combined with chemical reactions to transform surface of ZVI into iron nitrides and Fe–N_*x*_ coordination structures (Figure 2b and Table S1).^48–50,59,71^ In this process, ZVI powder is mixed with nitrogen-rich precursor(s), such as urea, melamine, or thiourea, sodium amide, and subjected to high-energy ball milling in a sealed, argon-filled jar containing steel balls.^48–50,59,61,71,72^ The high-speed rotation of the jar induces high-energy collisions and friction, facilitating thorough mixing and triggering chemical reactions between iron and nitrogen.^73,74^ This results in the intricate integration of nitrogen—sometimes accompanied by carbon from the nitrogen precursors such as melamine,^48,49^ urea,^49,71^ thiourea^71^—with the surface of iron particles. The final material may exhibit unique surface structures such as iron nitrides or surface-bound Fe–N_*x*_ coordination complexes, depending on the nitrogen precursors and milling conditions used.^48–50,59,71^

Several factors influence the efficiency and outcome of the mechanochemical nitridation process (Table S1). The selection of the solid nitrogen precursor is critical, as different nitridating agents behave differently under mechanical stress, affecting the final nitrogen content, speciation, and distribution. For instance, melamine and sodium amide used as nitrogen precursors resulted in the formation of Fe–N_*x*_ coordination structures^48,49^ and iron nitrides,^61^ respectively, on mechanochemically treated ZVI; while urea could lead to the formation of both iron nitrides and Fe–N_*x*_ coordination structures.^71^ The ratio of iron to nitrogen compound, size and number of milling balls (i.e., proportional to the energy input), as well as speed and duration of milling, also play crucial roles in determining the extent of nitridation and the properties of the synthesized N-ZVI as indicated by its reductive dechlorination reactivity. It was found that the dechlorination reactivity of N-ZVI increased when the ball milling speed was raised from 200 to 400 rpm, but further increasing it to 600 rpm only resulted in slightly higher activity compared to 200 rpm, indicating that excessive energy input reduced the material’s reactivity.^49^ Additionally, material properties of the milling container and balls can influence the nitridation efficiency and the characteristics of the final N-ZVI.^48,49,73–75^

### 2.2 Nitridation Mechanisms

#### 2.2.1 Nitridation through the Thermochemical Process

The preparation of N-ZVI via thermochemical treatment primarily involves nitrogen diffusion into the iron lattice under high-temperature conditions in a nitrogen-rich environment.^26,68^ This process progresses through two distinct stages: (i) nitrogen activation and initial interaction, and (ii) nitrogen diffusion and iron nitride formation, as illustrated in Figure 2a.

##### 2.2.2.1 Nitrogen Activation and Initial Interaction

Nitrogen activation and initial interaction mechanisms in gas–solid nitridation and solid–solid nitridation differ significantly due to variations in both nitrogen and iron precursors. In gas–solid nitridation, air-stable or pyrophoric (n)­ZVI particles (or almost any iron oxide/hydroxide powder) can be used as the iron precursor, with or without additional surface modification. Nitrogenous gases such as NH_3_ or mixtures of NH_3_ with N_2_ or H_2_ provide the gaseous nitrogen source; in the case of H_2_, it also acts as an additional reducing agent for the transformation of iron oxide/hydroxide precursors to (n)­ZVI. At temperatures typically from 300 to 600 °C, nitrogen-containing gases like NH_3_ adsorb onto the iron surface and dissociate into active nitrogen species^76^ (following the reaction 2NH_3_ → 2N + 6H → N_2_ + 3H_2_). Adsorption and dissociation of nitriding gases was suggested to be the rate-limiting step in gas–solid nitridation of nanocrystalline ZVI.^76^

In contrast, solid–solid thermochemical nitridation involves a more complex pathway where iron particles are embedded in a solid N-bearing precursor. For example, this method could begin with the preparation of an iron-ion-containing solution, typically derived from ferric chloride (or nitrate) or ferrous acetate, combined with a gel-forming agent such as a polymer or silica.^29,69,70^ A nitrogen source such as urea is then incorporated into the sol–gel matrix during gel formation or postgelation.^77,78^ Alternatively, the nitrogen source can be directly integrated into the gel-forming agent, e.g., by using gelatin.^29^ The gel is then dried to remove water and ground. Subsequent heat treatment under inert (N_2_) or reducing (N_2/_/H_2_) conditions results in nucleation of iron ions and formation of ZVI and/or iron oxide/hydroxide. Meanwhile, nitrogen species decompose and react with the iron (nano)­particle surface^29^ (Figure 2a).

##### 2.2.3.2 Diffusion and Formation of Iron Nitrides

Following surface adsorption, surface adsorbed nitrogen atoms diffuse deeper into the lattice of ZVI particles, driven by nitrogen partial pressure at elevated temperature.^79,80^ Within the metallic iron lattice, nitrogen occupies interstitial sites, modifying the electronic and structural properties of the material. Notably, although iron can remain in a metal-like state during the nitridation of nZVI, the integration of nitrogen or Fe_*x*_N and Fe^0^ does not form the preferred core–shell structure due to rapid N diffusion.^29^ Instead, nitrogen is uniformly embedded in the entire N-nZVI volume as a bulk Fe_*x*_N. The resulting N/Fe ratio and Fe_*x*_N phase composition are thermodynamically controlled by the nitriding potential (dictated by the pressure of the nitriding gas and its composition) and the achieved temperature.^81^

#### 2.2.2 Mechanism of Mechanochemical Nitridation

Mechanochemical nitridation enables the incorporation of nitrogen into ZVI structures, typically through high-energy ball milling under nitrogen-rich conditions. A key outcome of this process is the formation of Fe–N_*x*_ coordinated structures, particularly when using organic nitrogen-bearing precursors such as melamine,^48,49^ urea,^49,71^ and thiourea.^71^ The mechanochemical decomposition of these nitrogen-rich precursors generates reactive intermediates, including ammonia, biuret, cyanuric acid, and isocyanic acid, which play a crucial role in nitrogen incorporation (Figure 2b).^82^ Specifically, this process is characterized by the formation of defect-rich carbon structures resulting from the carbon present in organic precursors. These structures include pyridinic, pyrrolic, and graphitic nitrogen species,^83,84^ which readily coordinate with Fe^2+^ and Fe^3+^ of ZVI, leading to the formation of Fe–N_*x*_ moieties on the ZVI surface. This coordination is driven by the mechanical energy imparted during milling, which enhances atomic dispersion and promotes strong metal–ligand interactions (Figure 2b).^48,49^

Concurrent with the formation of Fe–N_*x*_ coordinated structure, Fe_*x*_N can also be formed during the mechanochemical nitridation process, depending on the specific conditions of the mechanochemical process and the choice of N precursors.^48,49^ Previous studies using Fourier-transform infrared spectroscopy (FTIR) have suggested a mechanism for the formation of Fe_*x*_N.^49,71^ During the decomposition of N/C-containing precursors (e.g., urea, thiourea), the N–H, N–C, and N=C bonds undergo cleavage under mechanochemical activation, leading to the formation of ^•^NH_2_ and/NH radicals.^49,85,86^ This process facilitates nitrogen incorporation into the iron lattice, mediated by transient localized heating generated during high-energy ball milling, ultimately driving Fe_*x*_N phase formation on the surface of ZVI particles.^49,85,86^

## 3 Physical Properties of N-ZVI

### 3.1 Microstructure and Morphology

Iron nitride (nano)­particles, prepared from (n)­ZVI precursor by thermochemical nitridation methods, typically exhibit a spherical morphology, which is similar to the (n)­ZVI precursor in the gas–solid process (Figure 3). Like (n)­ZVI precursor, N-(n)­ZVI particles are prone to aggregation. However, nitridation can lower particle aggregation depending on the details of the nitridation procedure. For instance, at low-to-high nitridation levels, N-nZVI samples produced via the gas–solid method exhibit spherical particles with compact oxide shells (Figure 3), formed during subsequent sample handling in air.^26^ The agglomerate size distribution of N-nZVI particles revealed a median agglomerate size of 1.6–2.6 μm, which was 20–50% less than that of the nZVI precursor.^26^ The solid–solid thermochemical nitridation method yields highly aggregated N-nZVI particles when the precursor is synthesized via the sol–gel method.^29^

**Figure 3:**
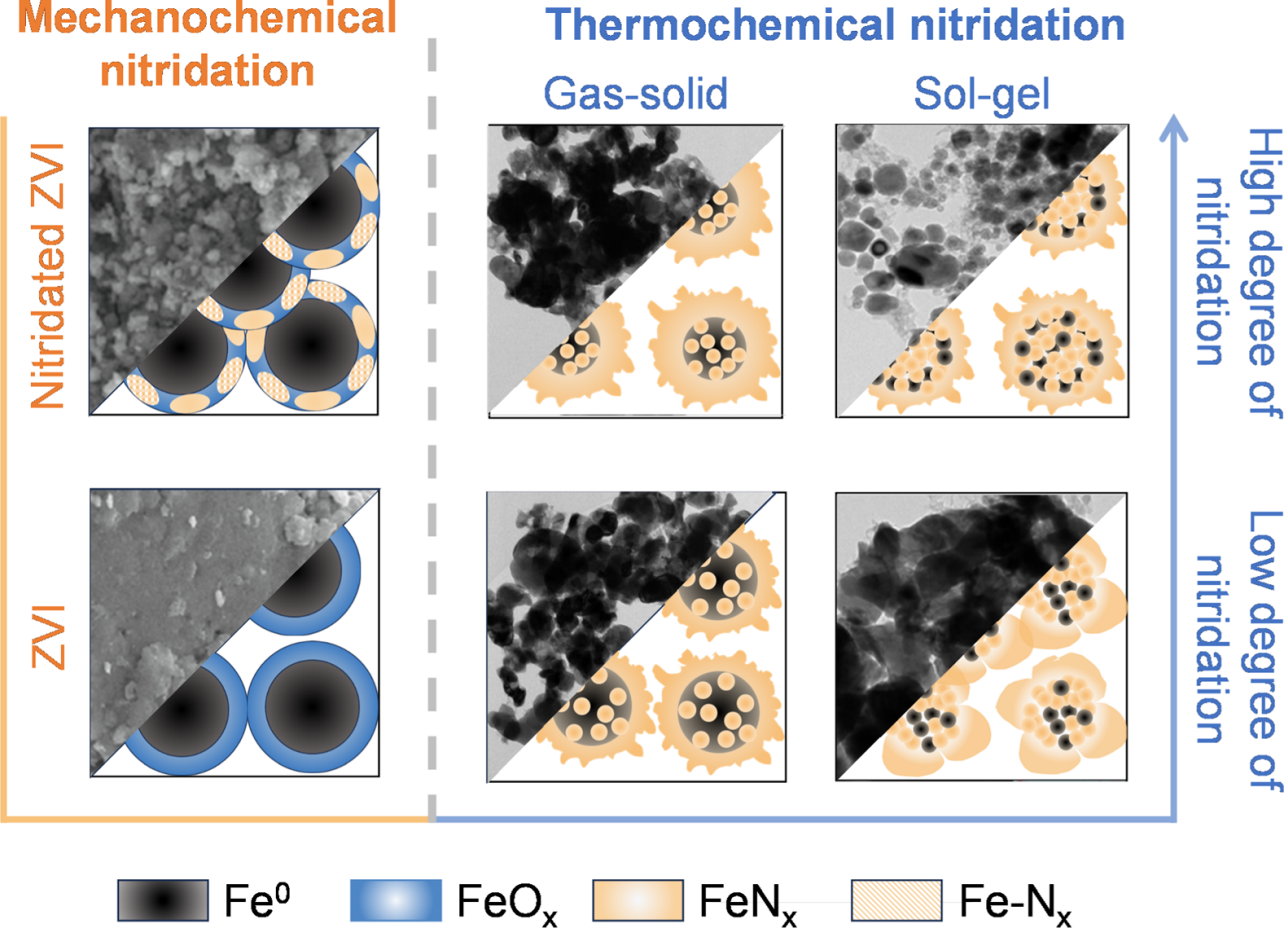
Schematic representations of N-ZVI morphology prepared via thermochemical nitridation methods and the mechanochemical ball milling nitridation method. The upper-left shows N-ZVI prepared by the mechanochemical method, while the lower-left shows unmodified ZVI. The lower-right shows N-ZVI with a low degree of nitridation obtained through thermochemical nitridation methods, whereas the upper-right presents N-ZVI with a high degree of nitridation prepared via thermochemical nitridation methods (this layout of this figure was adapted from a review S-ZVI^51^ to facilitate comparison between sulfidation and nitridation).

Microscale N-ZVI (N-mZVI) synthesized via mechanochemical ball milling exhibits morphological transformations, significantly influenced by the choice of nitrogen precursors and milling conditions. Non-nitridated microscale ZVI (mZVI) subjected to ball milling exhibits a flake-like morphology with minimal surface alterations.^48^ When dimethylimidazole (2-Melm) is employed as the nitrogen precursor, the surface of N-mZVI remains relatively unchanged, with negligible variations in particle size and specific surface area, closely resembling its non-nitridated counterpart.^49^ In contrast, employing urea and melamine as nitrogen precursors significantly increases the surface roughness of mZVI while promoting a pronounced reduction in particle size.^48,49^ These findings suggest that the selection of appropriate nitrogen precursors, i.e., mainly the chemical-bond properties contained in the nitrogen precursors, plays a crucial role in facilitating particle fragmentation during ball milling.^87^

### 3.2 Bulk Phase and Surface Composition of N-ZVI

Iron nitride phases have been consistently identified in (n)­ZVI particles nitridated via the thermochemical nitridation methods.^25–29^ In the gas–solid process, reaction temperature and nitriding potential (controlled by the pressure and NH_3_ content of the nitriding gas) play a critical role in determining the nitridation degree and phase composition. Nitridation at lower reaction temperatures necessitates a prolonged time for reaching the maximum (thermodynamically controlled) extent of nitridation. X-ray diffraction (XRD) analysis revealed that at lower temperatures (≤300 °C) with an NH_3_/N_2_ ratio of 2:1 in the nitriding gas, the dominant phase is ε-Fe_2–3_N.^26^ Conversely, increasing the reaction temperature (≥500 °C) shortens the nitridation time, favoring the formation of γ′-Fe_4_N as the predominant crystalline phase with an NH_3_/N_2_ ratio of 1:2.^26^ In contrast, ball milling introduces nitrogen interacting with the surface-exposed iron, primarily leading to the formation of Fe–N_*x*_ coordination structures.^48–50^ Existing studies indicate that Fe_*x*_N phases generated via mechanochemical nitridation are rarely detected by XRD, suggesting that the resulting iron nitrides are predominantly amorphous or of low crystallinity^71^ or present as a very thin layer (i.e., nondetectable by XRD).

A comparison of the N 1s X-ray photoelectron spectroscopy (XPS) spectra of N-ZVIs across different studies indicates that the choice of nitrogen precursors significantly influences the surface species and chemical states of N-ZVIs. When the nitrogen source lacks carbon, compounds such as Fe_*x*_N, oxidized Fe_*x*_N, and nitrogen oxides (NO_*x*_) are typically present on the N-nZVI surface prepared via the gas–solid thermochemical nitridation method.^26^ In contrast, nitrogen species such as pyridinic, pyrrolic, and graphitic nitrogen groups are identified when the nitrogen precursors contain carbon.^48,50,59^ For instance, in the precursor synthesized via the solid–solid method, the nitrogen precursor is gelatin, a nitrogen- and carbon-rich colloid. During high-temperature treatment, nitrogen reacts with iron to form the Fe_4_N phase,^29^ while carbon interacts with the iron surface to create three-dimensional network structures.^88^ Nitrogen further reacts with this network to generate Fe–N_*x*_. In mechanochemically synthesized N-mZVI using nitrogen/carbon containing precursors (e.g., urea,^49,71^ thiourea,^49,71^ or melamine^48,50^ ), mechanochemical energy induces precursor fragmentation, generating organic nitrogen intermediates that evolve into distinct coordination species (pyridinic-N, pyrrolic-N, and graphitic-N). Pyridinic/pyrrolic-N preferentially coordinate with surface Fe^2+^/Fe^3+^, driving Fe–N_*x*_ structure formation. Urea/thiourea decomposition under shear forces releases N radicals (^•^NH_2_, :NH), which react with atomic-scale defects on ZVI surfaces, leading to the formation of Fe_*x*_N phases.^71^ In contrast, melamine-derived N-mZVI exclusively forms Fe–N_*x*_.^48,50^

XPS Fe 2p spectra indicate that in N-nZVI particles prepared via the gas–solid thermochemical method, their surface exhibits a higher relative abundance of iron in reduced forms (either Fe^0^ or Fe_*x*_N) upon exposure to water compared to the precursor nZVI.^26^ For mechanochemically synthesized N-mZVI, the Fe 2p XPS generally reveals consistent spectral profiles with quantitative discrepancies in the peak intensities of Fe^0^, Fe^2+^, and Fe^3+^ among samples prepared using different N precursors. However, XPS lacks adequate resolution to clearly discriminate between metallic Fe^0^ and Fe_*x*_N species, as well as Fe^2+^, Fe^3+^, and Fe–N_*x*_ coordination structures due to overlapping binding energies.^48,50^

^57^Fe Mössbauer spectroscopy addresses this limitation by differentiating iron species with subtle electronic state variations,^89^ enabling more accurate identification of bulk iron species in N-ZVI. This technique not only enabled detection of Fe_*x*_N and Fe_*x*_O_*y*_ on the surface of N-nZVI synthesized via gas–solid methods (when Fe_*x*_O_*y*_ formed as an artifact of partial surface oxidation during sample preparation),^26^ but also revealed characteristic spectral features of Fe^0^ and Fe–N_*x*_ coordination structures in N-mZVI prepared by ball milling.^48^ FTIR and Raman spectroscopy can also be used to verify the presence of Fe–N_*x*_,^47^ although their applications are limited to surface chemical bonds, lacking effectiveness in analyzing the Fe_*x*_N phase.

The detailed material characterizations summarized above show that N-ZVI particles prepared by thermochemical methods could contain two forms of nonoxidized iron: (i) residual Fe^0^ from the ZVI precursor, and (ii) metallic-like iron in the form of Fe_*x*_N (i.e., with delocalized electrons among Fe atoms, similar to metallic bonding, and with partially covalent/partially metallic bonding between Fe and N). Fe^0^ content has been routinely determined for practical applications by measuring the amount of H_2_ generated from acid digestion^26,51,90–93^ provided that Fe_*x*_N can undergo hydrogen evolution reaction (HER).^29^ In general, Fe^0^ content in N-ZVI is affected by the synthesis method and is generally independent of the selection of nitrogen precursors. In N-nZVI synthesized via the gas–solid thermochemical nitridation method, Fe^0^ content exhibits an inverse correlation with the degree of nitridation: as the nitridation extent increases, residual Fe^0^ content decreases.^26^ This phenomenon arises from dissolving more electronegative nitrogen in metallic iron to form iron nitrides.^26^ For instance, while the precursor nZVI contained ∼80% of Fe^0^ per mass, the reducing capacity of γ′-Fe_4_N and ε-Fe_2–3_N prepared by the gas–solid method decreased by 33 and 75%, respectively.^26^ This seems to be an inherent limitation of the gas–solid method (at least for the nanoscale).

Unlike in the gas–solid nitridation method, an increased N/Fe molar ratio generally correlates with a higher Fe^0^ content in the solid–solid thermochemical nitridation method from sol–gel synthesized precursor. The Fe^0^ content in the particles can reach higher values (≥60%) at N/Fe molar ratios ≥0.07.^29^ This phenomenon is attributed to the role of nitrogen precursors in facilitating the nucleation of iron oxides/hydroxides and their subsequent conversion to Fe^0^ and Fe_*x*_N.^29^ Mechanochemical ball milling differs significantly from the aforementioned thermochemical nitridation methods in terms of Fe^0^ content. The Fe^0^ content in mZVI can be largely preserved (>90%) through ball milling-induced nitridation.^48,49^ Recent advancements have enabled the synthesis of sulfur- and nitrogen-modified ZVI (S–N-mZVI) via mechanochemical ball milling to further improve the N-mZVI reactivity with contaminants. This material retains a high Fe^0^ content (∼85%), along with significantly enhanced reactivity of N-mZVI compared to ball-milled N-mZVI without sulfur modification.^50,59,71^

### 3.3 Surface Hydrophobicity and Electrical Conductivity

The surface properties of ZVI are a major determinant of its chemical reactivity, including redox transformations and corrosion resistance. To elucidate the impact of nitridation on ZVI surface characteristics, the electrochemical properties and hydrophobicity of N-ZVI have been systematically investigated recently.^26,29,48,49^ The quantification of such properties is crucial in explaining the enhanced reactivity and electron efficiency of N-ZVI in pollutant transformation compared to unmodified counterparts.

Comparative analysis of N-ZVI synthesized via different nitridation methods reveals notable variations in surface hydrophobicity. N-nZVI prepared via the thermochemical nitridation methods exhibits increased hydrophobicity due to the presence of Fe_*x*_N (e.g., Fe_4_N) on the surface, which contrasts with the hydrophilic character of the thicker/extensive (oxyhydr)­oxide layer commonly found on unmodified nZVI.^26,29^ These findings align well with density functional theory (DFT) calculations that show decreased water affinity for nitrogen-doped iron surface and crystalline Fe_4_N compared to the pristine iron surface.^26,94^ Moreover, N-nZVI prepared by the solid–solid thermochemical method exhibits considerably higher hydrophobicity (with water contact angles up to 120°) than N-nZVI prepared by the gas–solid method, which is likely due to the presence of amorphous carbon formed from sol–gel precursor.^29^ Conversely, N-mZVI nitridated via ball milling with melamine exhibits reduced hydrophobicity due to the presence of polar nitrogen-functional groups, which interact with water molecules through hydrogen bonding and electrostatic interactions;^48,49^ while the hydrophobicity of N-mZVI synthesized using 2-Melm as the ball-milled nitrogen precursor remains relatively unchanged.^49^ The concurrent sulfur- and nitrogen-modification enhances the hydrophobicity of mZVI, further improving its selectivity for hydrophobic contaminants.^71^

Fe_*x*_N has relatively high electrical conductivity (2–3 × 10^3^ S m^–1^),^95^ which can mediate faster electron transfer, especially when compared with FeO_*x*_ (0–1 × 10^–1^ S m^–1^).^96^ This has been demonstrated by electrochemical impedance spectroscopy (EIS) and cyclic voltammetry (CV) performed on a three-electrode system,^29,49^ in which N-nZVI prepared via the thermochemical nitridation method was deposited at the glass carbon working electrode. For instance, EIS data show that the charge transfer resistance of N-nZVI is reduced by over 60% compared to unmodified nZVI, which favors faster electron transfer rates.^29^ The interplay between hydrophobic surface sites, electron conductivity as well as content of residual Fe^0^/iron nitride(s)/oxidized iron in N-ZVI must be considered in optimizing N-ZVI decontamination performance.

## 4 Effect of Nitridation on Pollutant Transformation

The mechanism of pollutant degradation by N-ZVI is highly contaminant-specific, governed by a complex interplay of adsorption and reduction processes, which are influenced by the physicochemical properties of both N-ZVI (bulk and surface) and the target contaminants under specific environmental conditions. Recent advancements in N-ZVI synthesis have enabled significant enhancement of its potential for pollutant remediation, particularly for the degradation of various chlorinated hydrocarbons under anoxic conditions.^25,26,29,48,59^ In this section, we focus on the degradation of chlorinated hydrocarbons by N-ZVI and critically examine how nitridation enhances its performance compared to conventional ZVI. By elucidating the underlying mechanisms, we aim to identify key factors—structural and surface modifications, electron transfer dynamics, and interfacial reactions—that contribute to improved pollutant elimination performance. This discussion provides a foundation for optimizing N-ZVI synthesis and expanding its practical applications in environmental remediation.

### 4.1 Treatment Performance Metrics

#### 4.1.1 Dechlorination Kinetics

Any treatment based on amendment with reactive materials must provide sufficiently high contaminant degradation rates to reach treatment goals. The rate of contaminant transformation by ZVI is most usefully represented by pseudo-first order rate constants that are normalized to ZVI mass (*k*_M_) or specific surface area (*k*_SA_),^97^ and simultaneous consideration of both these properties provides the most complete perspective on the relative reactivity of materials. This type of analysis has shown that rate enhancements from sulfidation of ZVI are not just due to increased specific surface area,^98^ and here we extend that analysis to consider nitridation. Figure 4a compares the distributions of *k*_M_ and *k*_SA_ for modified ZVIs from the ARK database vs the major categories of modified ZVIs.^99^ For the conventional types of ZVI and S-ZVI, there are many more data for a broader range of contaminants than for N-ZVIs. Currently, data for N-ZVI, ZVI, and S-ZVI are only available for trichloroethylene (TCE), chloroform (CF), tetrachloroethene (PCE), *trans*-1,2-dichloroethene (*trans*-DCE), 1,1-dichloroethene (1,1-DCE), *cis*-1,2-dichloroethene (*cis*-DCE), and vinyl chloride (VC). In general, particle size is expected to be a major determinant of differences in reactivity. However, for mZVIs, surface area normalization *k*_obs_ (*k*_SA_) does not substantially alter reactivity distributions versus mass-normalized rates (*k*_M_), indicating intrinsic reactivity dominates over surface area (SA) effects. For nZVIs, while *k*_M_ values are significantly higher than those of mZVIs due to increased surface area, *k*_SA_ values shift downward to levels comparable to mZVIs. This is consistent with prior work on the effects of sulfidation,^55^ and confirms that nanoscale enhancements primarily derive from increased SA rather than intrinsic reactivity. The new data for N-ZVIs reveal similar trends: SA normalization minimally shifts their distributions, and both *k*_M_ and *k*_SA_ align with unmodified mZVI. The available data show that *k*_M_ and *k*_SA_ for the ZVIs follow the order of untreated < nitridated < sulfidated ∼ nitridated + sulfidated, for both mZVI and nZVI.

**Figure 4:**
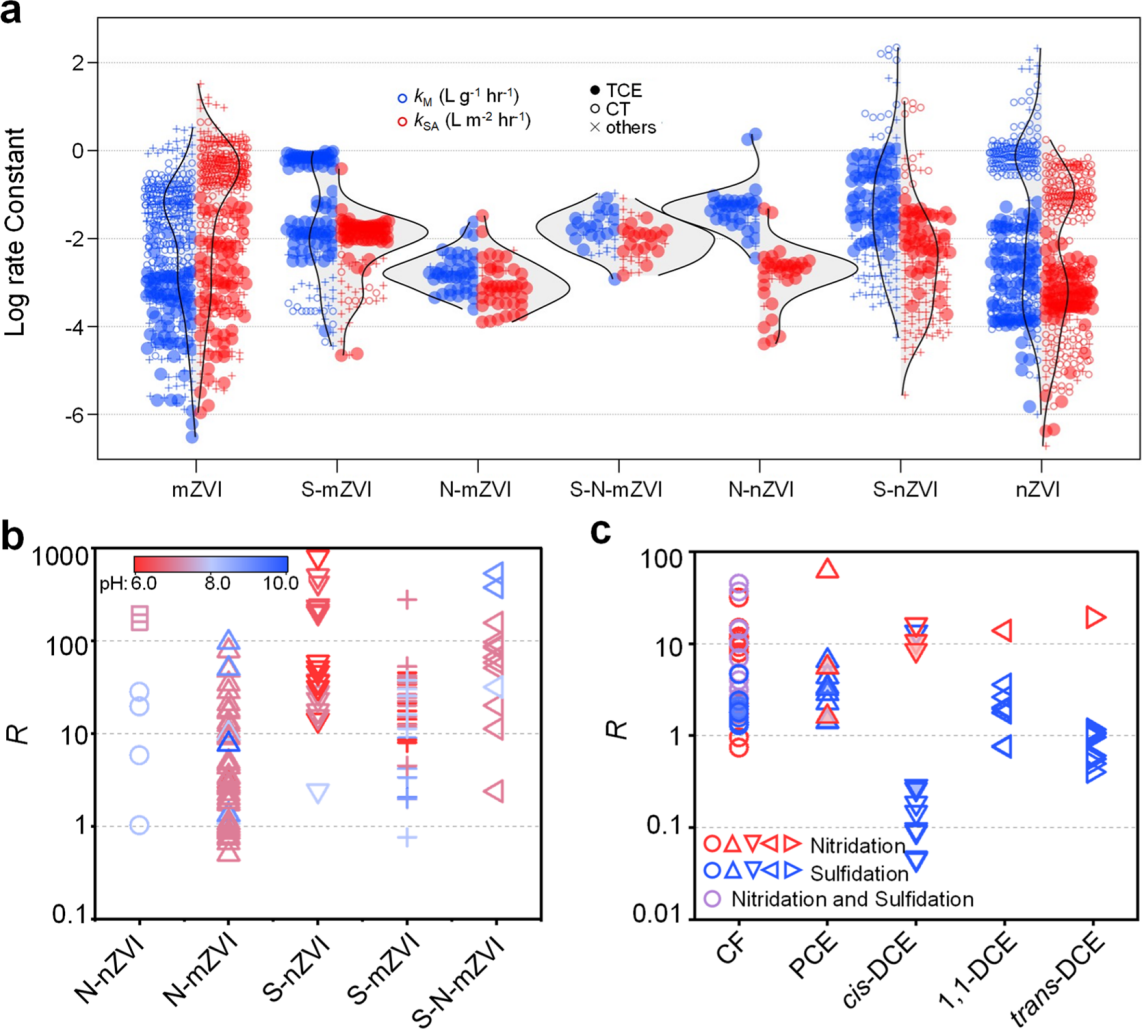
(a) Violin plot of log *k*_M_ (left) and log *k*_SA_ (right) of chlorinated hydrocarbon dechlorination by ZVIs, including N-ZVI, S-ZVI, and S–N-ZVI as well as unmodified ZVI in nanoscale (n) and microscale (m). Enhancement ratio (R) by *k*_M_ of N-ZVI, S-ZVI, and S–N-ZVI compared with unamended ZVI for (b) TCE (marker color represents pH) and (c) other chlorinated hydrocarbons (in panel “b”, the squares represent N-nZVI prepared by the solid–solid thermochemical nitridation method, and the circles represent N-nZVI prepared by the gas–solid thermochemical nitridation method). Data are from v1 of the ARK database.^98^

While Figure 4a provides a comprehensive perspective on the currently available data for key contaminants and all main types of ZVI, it confounds some of the operational variables that are known to affect contaminant reduction rates (pH, aging, cocontaminants, etc.), and this obscures some important effects of nitridation. To resolve these effects, Figure 4b,c show the relative value of rate constants for data that are available in pairs with and without treatment (i.e., *R* = *k*_M_^+treatment^/*k*_M_^–treatment^) vs material and contaminant type (Table S2). Values of *R* greater than 1 indicate that nitridation enhances reductive dechlorination by ZVI, whereas values below 1 suggest inhibition. Figure 4b shows that N-nZVI prepared via the gas–solid method exhibited considerable enhancements in TCE dechlorination rates,^26^ with an *R* value peaking at about 30. Similarly, the solid–solid thermochemical nitridation method achieved a notable enhancement ratio of about 130,^29^ although this value may be biased because the reference (non-nitridated) nZVI particles in that study were prepared using a different protocol. Moreover, the TCE degradation rate was slower under actual groundwater conditions, with complete dechlorination still observed within 48 h.

The enhancement of the TCE dechlorination rate by N-mZVI was more variable, with *R* values varying approximately between 1 and 100 depending on the pH of the reaction.^48^ Under neutral pH conditions, the *R* values remained low, ranging approximately between 1 and 4,^48,49^ indicating limited enhancement of the dechlorination rates. However, under alkaline conditions, where ZVI typically becomes passivated and therefore gives slower dechlorination kinetics, N-mZVI could maintain comparable dechlorination kinetics to that under neutral pH conditions, giving higher *R* values ranging approximately from 5 to 100. For instance, at pH levels of 8 to 9, the *R* value increased to about 100. N-ZVI maintains >80% reactivity at pH 9–10, whereas S-ZVI activity declines by 40–60% under identical conditions. While sulfidation also yields higher enhancement ratios for reductive dechlorination (Figure 4b,c), nitridation confers a distinct advantage in mitigating performance loss under alkaline conditions (Figure 4b). This enhanced resistance to pH effects suggests that nitridated surfaces are less susceptible to hydroxide-induced passivation compared to sulfidated or pristine ZVI. The defining trait of the Fe–N_*x*_ coordinated structure modified ZVI—alkaline resilience (>95% activity retention at pH 10)—is governed exclusively by Fe–N_*x*_ coordinated structures, as demonstrated by comparative analysis with carbon-modified ZVI.^100^

To further optimize the performance of N-mZVI, especially under neutral pH conditions, iron sulfide (FeS_*x*_) was introduced as an electron mediator^50,59,71^ to facilitate electron transfer from the Fe^0^ core to the particle surface. The resulting S–N-mZVI benefits from the synergistic effects of FeS_*x*_ and the Fe–N_*x*_, enabling efficient electron transfer to adsorbed TCE and optimizing dechlorination kinetics,^50,71^ which gives the higher *R* values (varying from about 5 to 530) than that of the N-mZVI (Figure 4b).

As shown in Figure 4b,c, nitridation influences the *R* values of nZVI and mZVI following the general order of TCE (*R* = 0.5–130) > PCE (*R* = 1–80) > CF (*R* = 1–25) > *cis*-DCE (*R* = ∼10). Notably, nitridation enhances dechlorination kinetics more than sulfidation for several common chlorinated contaminants, including CF, PCE, and *cis*-DCE (Figure 4c). This unique advantage over sulfidation highlights the potential of nitridation for enhancing the remediation of mixed chlorinated hydrocarbon contaminations using ZVI-based technologies. Figure 4c shows that S–N-mZVI gives an *R*-value close to 50 for CF degradation, significantly surpassing that of S-mZVI and N-mZVI.^59^ The *R* values higher than 10 for *cis*-DCE degradation using N-ZVI^25,61^ not only overcomes the inhibitory effects often observed with traditional S-ZVI^25,54,101^ but also highlight N-ZVI’s significant dechlorination reactivity with *cis*-DCE. The observed differences in R-values for N-ZVI and S-ZVI (calculated using *k*_M_) likely result from a more efficient electron transfer in N-ZVI systems and different activation and dechlorination pathways for these compounds. While the reactivity of N-ZVI is primarily controlled by direct electron transfer, as evidenced by the negative correlation between *R* and *E*_LUMO_ values,^61^ S-ZVI with lower sulfur loadings tends to favor H*-mediated pathways. This is supported by the weak correlations of *R* vs *E*_LUMO_ values.^102,103^ The mechanistic distinctions between N-ZVI and S-ZVI and the role of intrinsic chemical properties of the pollutants on the *R* values warrant further investigation.

In addition to the absolute (*k*_M_, *k*_SA_) and relative (*R*) rates of contaminant degradation, the utility of ZVI in practical treatment applications requires sustained reactivity over considerable time periods. This requirement is determined by four factors: (i) the “electron efficiency (ε_e_)”,^104^ which is the fraction of electrons from ZVI that is utilized for the reduction of contaminants rather than side reactions induced by natural reductant demand (NRD), most notably HER;^53^ (ii) the reducing capacity, which expressed the amount of reducing equivalents (e.g., electrons) available in the particles for reacting with contaminants and NRD; (iii) passivation of the ZVI where surface oxidation during particle aging reduces ZVI’s long-term reactivity;^105^ and (iv) distribution of reaction products, as the production of distinct hydrocarbons requires different numbers of electrons.

#### 4.1.2 Electron Efficiency

The electron efficiency (ε_e_) of ZVI is strongly influenced by competing side reactions (i.e., HER) that consume its reducing capacity without contributing to contaminant degradation.^104^ Nitridation has been demonstrated to enhance ε_e_, with the degree of improvement depending on the synthesis method.^26,29^ For example, N-nZVI synthesized by the latter method has an ε_e_ of 95%,^29^ which is remarkably higher than unmodified ZVI, which has an electron efficiency only of about 2–3% ^106,107^ (Figure 5 and Table S2). In contrast, N-mZVI shows limited improvement in ε_e_ compared to unmodified mZVI,^48,49^ as its dechlorination kinetics are enhanced without a proportional suppression of the HER.^50^ However, the ε_e_ of S–N-mZVI can be close to 50%, which is about 110 times higher than that of unmodified mZVI and 150 times higher than that of N-mZVI (Figure 5). It is worth noting that the increase in ε_e_ for S–N-mZVI is not only higher than that of N-mZVI, but also greater than most data for S-nZVI and S-mZVI.^50,71,108–112^

**Figure 5:**
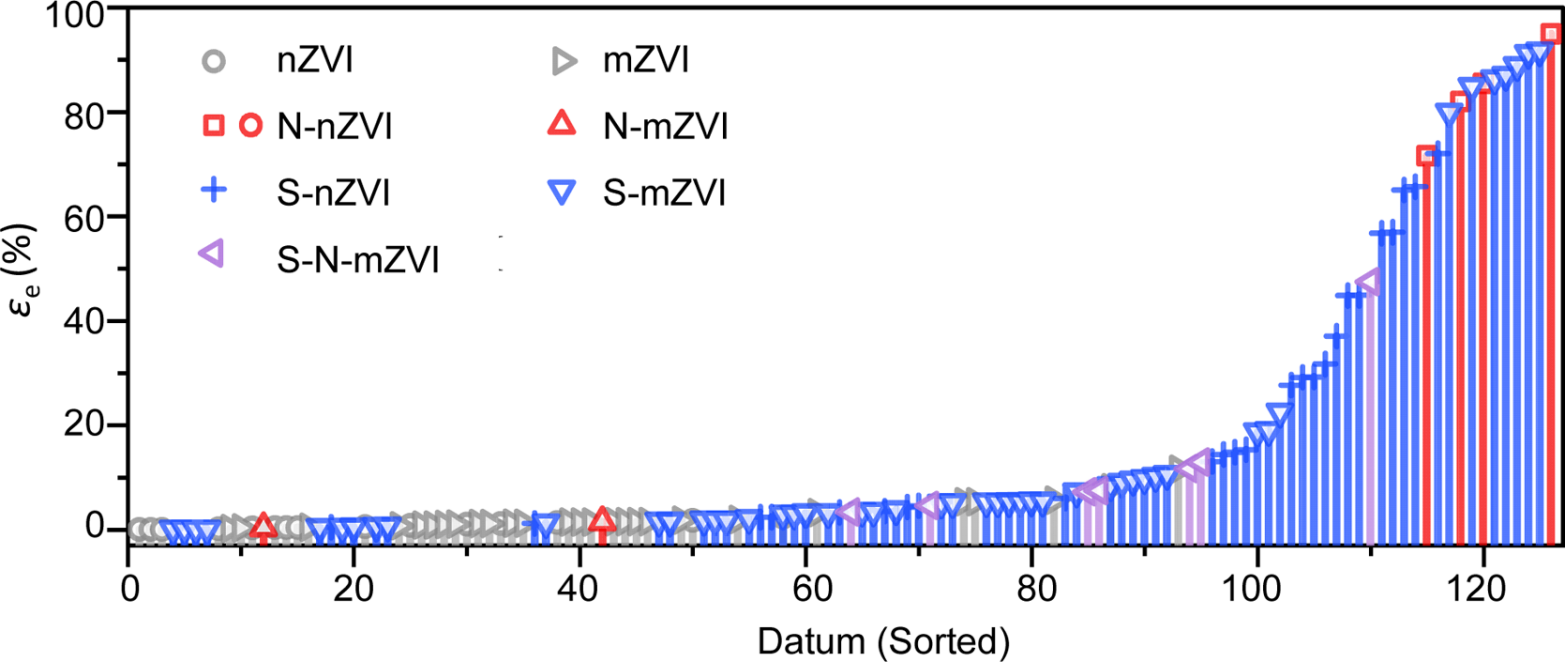
Summary of published electron efficiencies (ε_e_) from studies of chlorinated hydrocarbon reduction by ZVI. For each experiment, ε_e_ is plotted at one position on the datum axis, sorted by increasing ε_e_ value. Some of the main factors that affect the ε_e_ are represented in the figure by the marker type and color. The data and metadata compiled for this analysis are given in Table S2.^104^

#### 4.1.3 Reducing Capacity

The reducing capacity in ZVI-based systems is controlled mainly by the content of reduced iron (i.e., Fe^0^ or the equivalent in Fe_*x*_N)^55,113^ and therefore its preservation represents a crucial parameter for optimizing the synthesis procedure of any ZVI-based (nano)­materials. The Fe^0^ content of N-ZVI prepared using different nitridation methods has been discussed in detail in Section 3.2.

#### 4.1.4 Aging

The gradual decline in ZVI’s reactivity due to surface oxidation and transformations in the aqueous environment significantly affects its operational lifetime in environmental applications.^114–116^ Over time, Fe^0^ is oxidized to Fe^2+^/Fe^3+^, leading to the formation of Fe (oxyhydr)­oxides on the particle surface,^117^ which reduce its decontamination performance.

Crystalline Fe_*x*_N phases are known for their corrosion resistance, which directly enhances the resistance of N-ZVI to aging. Accordingly, N-nZVI composed mostly of γ′-Fe_4_N retained 57% of the initial reducing capacity after 104 days of aging, with a drop of only 60% in TCE dechlorination rate compared to fresh N-nZVI particles. In contrast, unmodified nZVI underwent surface passivation and its TCE dechlorination rate dropped by 75% under identical conditions.^26^ Meanwhile, N-nZVI composed predominantly of ε-Fe_2–3_N underwent limited corrosion and HER but exhibited a much faster decline in reactivity: a ∼20-fold reduction in TCE dechlorination rate after the same aging period. This behavior is attributed to the higher interstitial nitrogen content, which reduced the overall particle reducing capacity and ultimately led to complete oxidation despite the suppression of HER. These results suggest the existence of an optimal bulk nitridation level at which the N-ZVI particles exhibit enhanced reactivity and electron selectivity toward contaminants while maintaining sufficient reducing capacity.

The corrosion resistance of N-ZVI with a low degree of nitridation appears comparable to that of S-nZVI, which showed a decline in reducing capacity of about 50% after 120 days of aging.^118^ However, the S-nZVI aging experiments were conducted under static conditions that minimize aging effects, while the N-nZVI aging experiments were performed under dynamic (shaking) conditions.^26^ This implies that the lifespan of N-nZVI with an appropriate extent of nitriding may surpass the longevity of S-nZVI under comparable conditions.

In contrast to ZVI, S-ZVI, and N-ZVI, the effect of S–N-mZVI aging appears minimal. After 10 days of aging, the rate of CF dechlorination by S–N-mZVI was nearly unchanged, while mZVI’s dechlorination kinetics decreased by about 30%.^59^ This greater stability of S–N-nZVI must be due to some synergistic effects of sulfidation and nitridation, perhaps because aging converts the Fe^2+^/Fe^3+^ oxides on the surface of S–N-mZVI to Fe_3_O_4_ (via the Schikorr reaction),^59^ which is the most stable iron oxide with high conductivity.^34^

Aging experiments conducted in the presence of common groundwater solutes^27^ demonstrated that N-nZVI maintains high reactivity with TCE after one month of exposure to Na^+^, Ca^2+^, Mg^2+^, Cl^–^, SO_4_^2–^, and HCO_3_^–^ solutions, confirming its strong potential for effective decontamination under realistic environmental conditions. The TCE degradation was strongly suppressed only in the presence of HPO_4_^2–^ and NO_3_^–^ ions. Previous research indicates that S-nZVI particles may be more prone to surface passivation in HPO_4_^2–^-rich waters, compared to N-nZVI, yet exhibit lower susceptibility to passivation in NO_3_^–^-impacted waters.^119^ Nevertheless, these comparisons are based on studies employing different methodologies. To accurately assess the relative longevity of N-ZVI, S-ZVI, and S–N-ZVI particles and the impact of groundwater solutes on their corrosion rate and reactivity, systematic comparative aging studies using consistent experimental protocols are needed.

The fate of nitrogen in N-ZVI particles during aging is controlled by its content and speciation.^26,59,61^ Fe–N_*x*_ moieties formed by the mechanochemical synthesis transform from pyridinic N to pyrrolic and graphitic N, while nitrogen leaching is negligible (<0.1 mg/L), which is most likely due to the stable Fe–N covalent bonding.^48,50^ Negligible leaching of N was also found for N-ZVI in short-term experiments (48 h).^61^ In contrast, the transformations of Fe_*x*_N phases lead to a gradual release of nitrogen in the form of ammonia in long-term tests (>100 days).^120^ While the leaching of ammonia could be problematic in surface water treatment, its continuous release at low levels to groundwater could also increase the efficiency of combined abiotic–biotic treatments by providing an exogenous nitrogen source, stimulating reductive dechlorination by microorganisms.^120^ The amount of leachable nitrogen can be minimized by controlled N-ZVI synthesis to obtain Fe_*x*_N phases with high Fe, low N stoichiometry, and/or by creating Fe_*x*_N phases only on the particle surface.

### 4.2 Mechanisms of Contaminant Reduction by Nitridated ZVI

The nitridation of ZVI enhances its ability to reduce contaminants, as particularly observed for dechlorinating chlorinated hydrocarbons. These advantages stem from multiple factors. First, nitridation alters the electron transfer capability of ZVI, enhancing electron transfer from its Fe^0^/Fe_*x*_N core to its surface and increasing the surface electron availability for reductive transformations. Second, nitridation modifies the ZVI-water interface, which affects not only the adsorption and transformation of contaminants on its surface, but also competing reactions on the surface, like HER.^25,26,29,48,50,53,71,121–123^

#### 4.2.1 Adsorption and Activation

Dechlorination via N-ZVI progresses through three phases: (i) adsorption of chlorinated hydrocarbons onto the N-ZVI surface, (ii) surface-mediated reactions, and (iii) product desorption. DFT studies revealed the mechanism of the dechlorination of chlorinated ethenes at the atomic level.^25,26,61^ Initially, chlorinated ethenes physically adsorb onto the N-ZVI surface, where its adsorption is influenced by the level of chlorination: compounds with higher chlorine content typically exhibit more favorable adsorption energies.^25,26^ Nitridation of nZVI enhances this step by increasing surface hydrophobicity and protecting it from oxidation, thereby increasing the contaminant adsorption capacity and the surface reactivity.^26^ In the next step, the physically adsorbed chlorinated ethenes can transition into a chemisorbed state.^25,121,122^ This chemisorption step activates the C–Cl bonds, facilitating their cleavage (Figure 6a). Iron nitride surfaces, in particular the γ′-Fe_4_N­(001) surface, reduce C–Cl bond dissociation energy barriers of TCE by nearly 15-fold compared to the isolated TCE molecule.^26^

**Figure 6:**
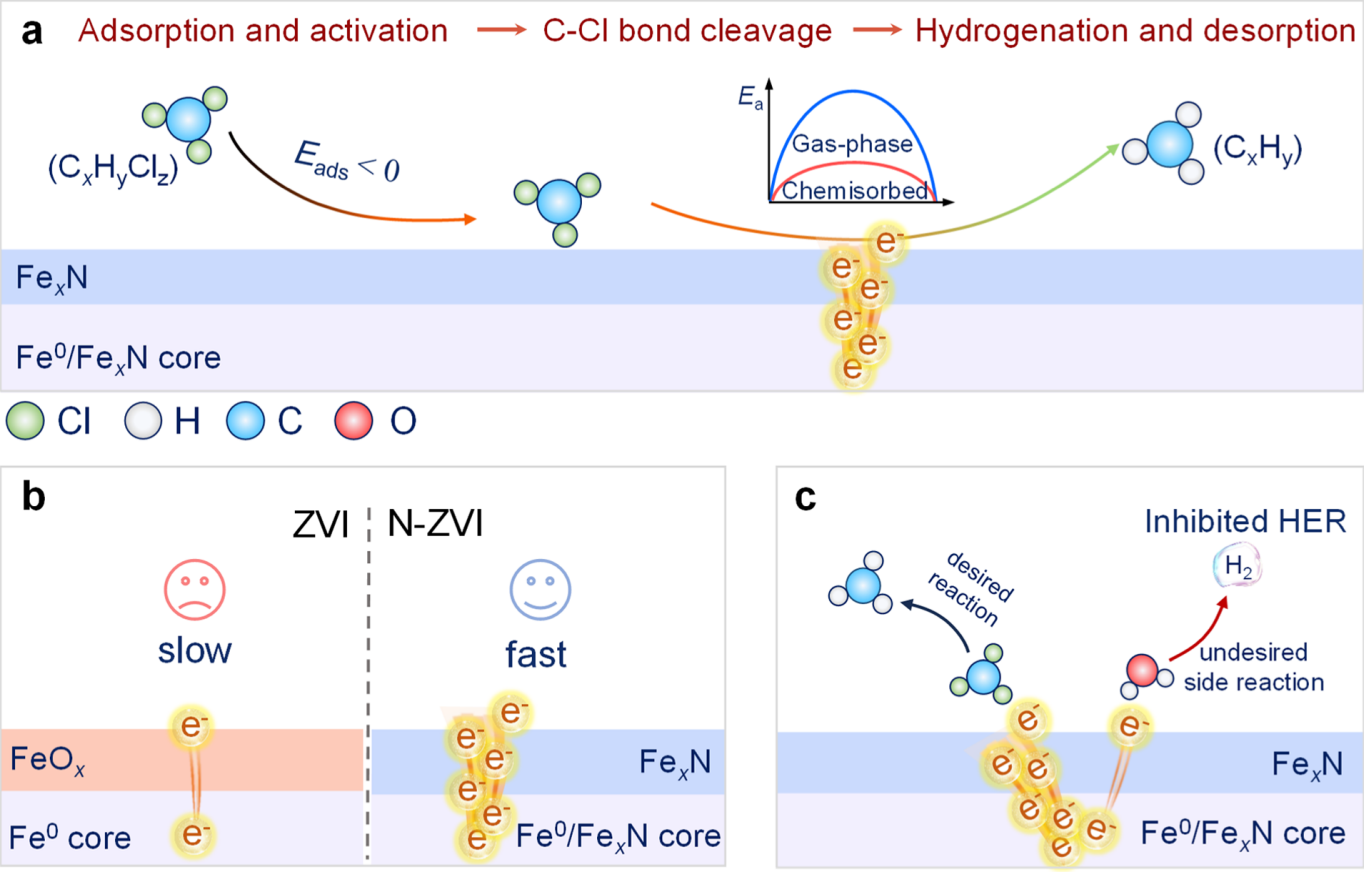
Mechanism of enhanced reductive dechlorination by N-ZVI: (a) adsorption and activation of chlorinated carbons by iron nitrides in reductive dechlorination by N-ZVI, (b) enhanced electron transfer from the Fe^0^/Fe_*x*_N core to the surface of N-ZVI due to increased electron conductivity of Fe_*x*_N compared with FeO_*x*_, (c) inhibition of the hydrogen evolution side reaction caused by Fe_*x*_N.

#### 4.2.2 Electron and Proton Transfer

The fast electron transfer rate of N-nZVI discussed in Section 3.3 affords rapid dechlorination kinetics observed for the N-nZVI prepared via thermochemical nitridation methods. Conversely, while the Fe–N_*x*_ in N-mZVI has some limitations in improving its electron transfer properties, Fe–N_*x*_ can significantly promote interfacial proton transfer (Figure 6b),^48^ and thus reduce the energy barrier of proton transfer involved in the reductive dechlorination reaction. This effect is somewhat limited for Fe–N_*x*_ alone; however, when combined with sulfidation (S–N-mZVI), the synergy between Fe–N_*x*_ and FeS_*x*_ facilitates both electron and proton transfer, resulting in up to approximately 135-fold and 45-fold increases in TCE and CF degradation rates, respectively, compared to unmodified ZVI.^50,59,71^

The underlying mechanism controlling the different reactivity trends observed among structurally similar chlorinated hydrocarbons for N-ZVI and S-ZVI, as discussed in Section 4.1.1, is not entirely clear. DFT calculations suggest that the energy barriers for dechlorination of PCE, TCE, and *cis*-DCE at the γ′-Fe_4_N surface are closer to each other compared to those at sulfidated iron surfaces, where electron transfer to *cis*-DCE is relatively less favored and PCE dechlorination can be hindered by steric effects of S atoms, leading to the preference of S-nZVI for TCE dechlorination.^25,124^ However, directly probing these surface-mediated mechanisms for volatile chlorinated organics under aqueous, anoxic conditions remains experimentally difficult, resulting in limited application of methods such as in situ Raman and ATR-FTIR, which have been primarily used to study nonvolatile pollutants or material characterization. To fully elucidate the factors responsible for the greater versatility of N-ZVI in the removal of mixed chlorinated pollutants, further experimental mechanistic investigations using the combination of electrochemical, isotope-specific, and quenching experiments are required.

#### 4.2.3 Hydrogenolysis, Hydrogenation, and Coupling Reactions

The intermediates formed by the initial reduction by direct electron transfer can further undergo various reactions, such as hydrogenolysis (i.e., replacement of a Cl atom by hydrogen), hydrogenation, and coupling reactions, which influence product distribution. The distribution of dechlorination products in N-ZVI is closely intertwined with its surface chemistry.

Owing to their high conductivity, Fe_*x*_N phases initially favor the production of acetylene from chlorinated ethenes by β-elimination.^25,26,29^ Acetylene can further undergo hydrogenation to form ethene and ethane, as well as coupling reactions producing hydrocarbons with longer carbon chains.^33,57^ N-nZVI prepared by the gas–solid thermochemical method yielded a higher fraction of hydrogenation and coupling products than N-nZVI prepared by the solid–solid thermochemical method (i.e., with a sol–gel synthesized precursor), where acetylene was the dominant product. This discrepancy may be explained by two factors: (i) acetylene reactions were inhibited by the latter N-nZVI type due to the presence of amorphous C/iron carbides on its surface; or (ii) the reaction time (8 h) was not sufficient to obtain stable product distribution, as in the study with the former N-nZVI type (21 days). Interestingly, the presence of coupling products with an odd number of carbon atoms suggests that carbon–carbon bonds can be cleaved during reductive dechlorination of chlorinated ethenes at Fe_*x*_N surfaces (Figure 6c).^25,26^ The resulting coupling of methane radicals likely proceeds through Fischer–Tropsch-type reactions, which are catalyzed by Fe_*x*_N species.^125^ N-mZVI prepared by the mechanochemical method shows limited impact on TCE degradation products by ZVI.^48,49^ When combined with FeS_*x*_ to form S–N-mZVI, acetylene reduction was inhibited and it was accumulated during TCE dechlorination, which is in line with the suppressed acetylene hydrogenation observed previously in S-ZVI systems.^50,57,90^

The saturation of dechlorination products controls the number of electrons required to degrade chlorinated hydrocarbons. For instance, the reduction of 1 mol of TCE to acetylene requires just 4 mol of electrons, far fewer than the 6 mol needed for ethene and the 8 mol for ethane. Consequently, nitridation strategies, which steer the reaction selectivity toward less saturated products like acetylene, can effectively minimize the number of reducing equivalents needed for the degradation of chlorinated hydrocarbons by N-ZVI.

#### 4.2.4 Side Reaction of the Hydrogen Evolution Reaction

HER competes with dechlorination for electrons, decreasing both the rate and (electron) efficiency of dechlorination. Nitridation overcomes this issue by increasing the hydrophobicity of the ZVI surface through the gradual formation of Fe_*x*_N and, in the case of the solid–solid thermochemical method, also amorphous carbon materials.^104^ This higher surface hydrophobicity minimizes water adsorption, suppressing HER and directing more electrons toward reactions with adsorbed contaminants. Experimental studies have shown that HER rates on N-nZVI can be decreased by approximately 70% compared to unmodified ZVI,^26^ minimizing electron competition between chlorinated hydrocarbons and water molecules, and leading to improvement of N-ZVI’s reductive dechlorination kinetics. In contrast, Fe–N_*x*_ coordination structures have a minimal impact on hydrophobicity and do not significantly suppress HER,^48^ and thus have minimal impact on the N-mZVI electron efficiency and particle longevity.

## 5 Environmental Implications and Future Perspectives

Nitridation has recently emerged as a new strategy to enhance the reactivity, electron efficiency, and long-term stability of ZVI for environmental applications. While sharing many benefits with the state-of-the-art sulfidation approach, nitridation offers unique advantages. This review summarizes the latest advances in N-ZVI, including the synthesis, characterization, and environmental remediation performance of nitridated iron-based (nano)­materials. The synthesis methods, whether thermochemical or mechanochemical, directly control the formation of active phases (e.g., Fe_*x*_N, Fe–N_*x*_), which in turn dictates the degradation performance and mechanisms. Nitridation of ZVI can improve its remediation performance as N-ZVI has significant advantages, including enhanced increasing dechlorination rates and electron efficiency by suppressing the HER. In particular, it exhibits superior dechlorination rate against prevalent pollutants (e.g., PCE, *cis*-DCE) and outperforms S-ZVI in degrading others (e.g., *cis*-DCE, CF). Mechanistically, the Fe_*x*_N phases are pivotal for enhancing electron transfer and imparting corrosion resistance, thereby extending the reactive lifespan, while Fe–N_*x*_ coordination structures facilitate distinct proton transfer. To advance N-ZVI technology toward practical environmental applications, several key knowledge gaps and challenges need to be addressed.

### Fundamental Understanding of Nitridation Mechanisms and Precision Synthesis of N-ZVI

The choice of nitridation methods, along with nitrogen precursors and process conditions, determines the structural and functional properties of N-ZVI. The foremost priority is the precision synthesis of N-ZVI with a core–shell Fe^0^–Fe_*x*_N structure, which are anticipated to combine prolonged reactivity with minimized ammonia leaching. This necessitates fundamental investigations into the kinetics of nitrogen precursor dissociation, nitrogen diffusion, and Fe_*x*_N phase formation during thermochemical nitridation under varied thermal conditions, alongside establishing quantitative relationships between mechanochemical parameters and the resulting Fe_*x*_N/Fe–N_*x*_ phase distribution. Exploratory work could also assess other nitridation methods like cold plasma treatment, which may yield unique N-ZVI compositions with tailored performance.

### Mechanistic and Comparative Reactivity Studies

In-depth mechanistic investigations are required to clarify the factors controlling the reactivity patterns of N-ZVI with different contaminants. Comparative studies assessing the reactivity and long-term performance studies with S-ZVI and other benchmark materials under varied environmental conditions and pollutant compositions are also crucial for understanding their relative performance.

### Expansion of Application Scope

Future research should extend N-ZVI applications beyond groundwater remediation to include waste- and drinking-water treatment, as well as soil decontamination. The potential of N-ZVI for eliminating recalcitrant contaminants, such as per- and polyfluoroalkyl substances (PFAS), antibiotics, and heavy metals, also merits detailed investigation. Combining N-ZVI with other remediation techniques could yield more robust and versatile environmental remediation strategies. The low concentrations of nitrogen leaching from the Fe_*x*_N materials could serve as a nitrogen source for dehalogenating bacteria. Additionally, coupling N-ZVI with advanced oxidation processes is a promising direction for future research, given their superior electron transfer and antipassivation properties.

### Sustainable Production and Life-Cycle Assessment

From a material engineering perspective, comprehensive life-cycle assessment (LCA) studies are needed to evaluate the holistic environmental impacts of N-ZVI. Future LCA studies should prioritize several key factors, including the environmental footprint of different nitrogen precursors, the energy intensity of various synthesis methods, and the long-term fate of nitrogen species in N-ZVI and environmental matrices. Particular attention should be given to potential trade-offs between enhanced treatment performance and possible secondary impacts such as nitrogen release. These assessments should be combined with the development of sustainable, low-carbon production methods that are economically viable, which is crucial for the large-scale application of these materials. These studies are indispensable to ensure that the enhanced remediation performance does not come at the expense of secondary pollution (e.g., through evolving ammonia).

## Supplementary Material



## Notes

Additional information on nitridation methods, and dechlorination kinetics of chlorinated carbons by N-ZVI (PDF)
