# Supplementary material for: Nitridated Iron-Based (Nano)Materials for Environmental Remediation: Synthesis, Characterization, and Performance
Source: Environ Sci Technol. 2025 Nov 14;59(46):24634–49. doi: 10.1021/acs.est.5c10601 (PMC12659439; doi:10.1021/acs.est.5c10601)
Supplement: Supplementary file 1 [file es5c10601_si_001.pdf]

Supporting Information

Nitridated Iron-Based (Nano)Materials for Environmental Remediation: Synthesis, Characterization, and Performance

Li Gong[1*], Jingting Chen[1], Feng He[1, 2], Miroslav Brumovský[3], Jan Filip[3*], Paul G. Tratnyek[4*]

[1]Zhejiang Key Laboratory of Low-carbon Control Technology for Industrial Pollution, College of Environment, Zhejiang University of Technology, Hangzhou 310014, China

[2]School of Environment and Ecology, Jiangnan University, Wuxi 214122, China

[3]Regional Centre of Advanced Technologies and Materials, Czech Advanced Technology and Research Institute, Palacký University Olomouc, CZ-78371 Olomouc, Czech Republic

[4]OHSU-PSU School of Public Health, Oregon Health & Science University, Portland, Oregon, 97239, United States

Corresponding authors: glili@zjut.edu.cn; jan.filip@upol.cz; tratnyek@ohsu.edu.

Keywords: Thermochemical nitridation, Mechanochemical nitridation, Zero-valent iron, Iron nitrides, Fe-N coordination structures

Summary

Number of pages: 18

Number of tables: 2

Contents

Table S1 .... S3
Table S2 .... S4
References S17

Table S1. Methods of nitridation of iron-based materials.

Nitridation method		Nitridation conditions				Final N speciation	Reference
		N source	Iron precursor	Temperature / speed of milling	Duration		
Thermochemical nitridation	Gas-solid	NH_3/N_2 (1:2)	nZVI	500 ℃	3 h	γ′-Fe_4N	Brumovsky/Filip[1]
		NH_3/N_2 (2:1)		300 ℃	5.5 h	ε-$Fe_{2\text{-}3}N$	
		NH_3/N_2 (1:1)		430 ℃	3.5 h	γ′-Fe_4N	Oborna/Filip[2]
	Solid-solid	gelatin	$C_4H_6FeO_4$	650 ℃	3 h	Fe_4N	Meng/Xu[3]
Mechanochemical nitridation	Solid-solid	urea, melamine, thiourea (in Ar)	Fe powder	200-600 rpm	5-20 h	Fe_xN and/or Fe–N_x coordination complexes on ZVI surface	Gong/He[4, 5]
		$NaNH_2$	Fe powder, Fe_3O_4			Fe_xN on ZVI surface	Gong/Zhang[6]

Table S2. Summary of rate constants (k_{obs}, k_M, and k_{SA}) and electron efficiency (ε_e) from relevant literature for chlorinated hydrocarbons dechlorination by sulfidated and nitridated ZVIs under similar conditions. Adapted from ARK[7] for use in Figures 4 and 5.

Primary Modification (Ligand)	Modification Method	Modification Dose	Fromed N species	Fe^0 content*	Target	pH initial	k_{obs} (1/hr)	k_M (L/h/g)	k_{SA} (L/h/m^2)	Electron Efficiency (%)	enhancement ratio (R) of k_M	Data Source
mZVI	Fe powder, ball milled				TCE	7.00	0.013	0.001	0.001	1.19		
			Fe-N_x			7.00	0.140	0.014	0.033	1.21		Shi/ Tang[8]
N, B-mZVI	Ni, $C_3H_6N_6$, Fe powder, Ball milled	N/Fe = 0.05	Fe-N_x		TCE	7.00	0.056	0.006	0.004	6.21		
			Fe-N_x			7.00	0.120	0.012	0.004	8.90		
mZVI	Fe powder, ball milled				TCE	7.00	0.007	0.001	0.000			
	Fe powder, $C_5H_8N_2$, Ball milled				TCE	7.00	0.004	0.000	0.000		0.66	
	Fe powder, CH_4N_2O, Ball milled				TCE	7.00	0.014	0.001	0.000		2.04	
			Fe-N_x			7.00	0.023	0.002	0.001		3.46	
		N/Fe = 0	Fe-N_x			7.00	0.006	0.001	0.000		0.86	
		N/Fe = 0.025	Fe-N_x			7.00	0.007	0.001	0.000		1.10	
		N/Fe = 0.05	Fe-N_x			7.00	0.013	0.001	0.000		2.00	Shi/ Tang[9]
N-mZVI		N/Fe = 0.1	Fe-N_x			7.00	0.005	0.001	0.000		0.78	
	Fe powder, $C_3H_6N_6$, Ball milled	N/Fe = 0.2	Fe-N_x		TCE	7.00	0.005	0.000	0.000		0.74	
			Fe-N_x			7.00	0.007	0.001	0.000		1.01	
			Fe-N_x			7.00	0.018	0.002	0.000		2.69	
			Fe-N_x			7.00	0.033	0.003	0.001		4.95	
		N/Fe = 0.05	Fe-N_x			7.00	0.012	0.001	0.000		1.84	
			Fe-N_x			7.00	0.029	0.003	0.001		4.34	
			Fe-N_x			7.00	0.016	0.002	0.000			
				91.30	TCE	7.00	0.003	0.000	0.001	0.043		
				91.30	*trans*-DCE	7.00	0.018	0.002	0.004			
				91.30	*cis*-DCE	7.00	0.019	0.002	0.004			
mZVI	Fe powder, Ball milled			91.30	1,1-DCE	7.00	0.001	0.000	0.000			
				91.30	VC	7.00	0.014	0.001	0.003			
				91.30	PCE	7.00	0.001	0.000	0.000			
				91.30	CF	7.00	0.001	0.000	0.000			
		Fe_3O_4/Fe = 0.11		78.90		7.00	0.009	0.001				
		Fe_3O_4/Fe = 0.17		73.60		7.00	0.009	0.001	0.001	0.86		Gong/ Zhang[6]
		Fe_3O_4/Fe = 0.2		68.00	TCE	7.00	0.009	0.001				
		Fe_3O_4/Fe = 0.33		57.60		7.00	0.012	0.001				
	Fe powder, Fe_3O_4, Ball milled	Fe_3O_4/Fe = 1		27.50		7.00	0.001	0.001				
mZVI		Fe_3O_4/Fe = 0.11		78.90		7.00	0.009	0.001				
		Fe_3O_4/Fe = 0.17		73.60		7.00	0.019	0.002	0.018			
		Fe_3O_4/Fe = 0.2		68.00	CF	7.00	0.007	0.001				
		Fe_3O_4/Fe = 0.33		57.60		7.00	0.011	0.001				
		Fe_3O_4/Fe = 1		27.50		7.00	0.016	0.001				

Primary Modification (Ligand)	Modification Method	Modification Dose	Fromed N species	Fe^0 content*	Target	pH initial	k_{obs} (1/hr)	k_M (L/h/g)	k_{SA} (L/h/m^2)	Electron Efficiency (%)	enhancement ratio (R) of k_M	Data Source
mZVI	Fe powder, Fe_3O_4, Ball milled	Fe_3O_4/Fe = 0.17		73.60	*trans*-DCE	7.00	0.032	0.003	0.003			Gong/ Zhang [6]
				73.60	*cis*-DCE	7.00	0.026	0.003	0.003			
				73.60	1,1-DCE	7.00	0.014	0.001	0.001			
				73.60	VC	7.00	0.027	0.003	0.003			
				73.60	PCE	7.00	0.008	0.001	0.000			
N-mZVI	Fe powder, $NaNH_2$, Ball milled	N/Fe = 0.1	Fe_xN	76.5	TCE	7.00	0.017	0.002			4.5	
	Fe powder, Fe_3O_4, $NaNH_2$, Ball milled	N/Fe = 0.1, Fe_3O_4/Fe = 0.17	Fe_xN			7.00	0.035	0.004			9.2	
		N/Fe = 0.1, Fe_3O_4/Fe = 0.33	Fe_xN			7.00	0.038	0.004			9.8	
		N/Fe = 0.1, Fe_3O_4/Fe = 1	Fe_xN	19.7		7.00	0.066	0.007			17.05	
		N/Fe = 0.2, Fe_3O_4/Fe = 0.11	Fe_xN	72.40		7.00	0.018	0.002			4.73	
		N/Fe = 0.2, Fe_3O_4/Fe = 0.17	Fe_xN	68.70		7.00	0.036	0.004			9.30	
		N/Fe = 0.2, Fe_3O_4/Fe = 0.2	Fe_xN	65.30		7.00	0.039	0.004			10.08	
		N/Fe = 0.2, Fe_3O_4/Fe = 0.33	Fe_xN	55.50		7.00	0.069	0.007			17.83	
		N/Fe = 0.2, Fe_3O_4/Fe = 1	Fe_xN	16.80		7.00	0.132	0.013			34.11	
		N/Fe = 0.3, Fe_3O_4/Fe = 0.17	Fe_xN	70.60		7.00	0.302	0.030	0.008	8.25	77.91	
		N/Fe = 0.3, Fe_3O_4/Fe = 0.33	Fe_xN			7.00	0.109	0.011			28.14	
		N/Fe = 0.3, Fe_3O_4/Fe = 1	Fe_xN	16.70		7.00	0.081	0.008			20.93	
		N/Fe = 0.4, Fe_3O_4/Fe = 0.17	Fe_xN	74.20		7.00	0.041	0.004			10.65	
		N/Fe = 0.4, Fe_3O_4/Fe = 1	Fe_xN	22.90		7.00	0.048	0.005			12.40	
		N/Fe = 0.5, Fe_3O_4/Fe = 1	Fe_xN	21.90		7.00	0.05	0.005			13.17	
	Fe powder, $NaNH_2$, Ball milled	N/Fe = 0.1	Fe_xN	76.5	CF	7.00	0.010	0.001			1.54	
	Fe powder, Fe_3O_4, $NaNH_2$, Ball milled	N/Fe = 0.1, Fe_3O_4/Fe = 0.17	Fe_xN			7.00	0.055	0.005			8.46	
		N/Fe = 0.1, Fe_3O_4/Fe = 0.33	Fe_xN			7.00	0.044	0.004			6.73	
		N/Fe = 0.1, Fe_3O_4/Fe = 1	Fe_xN	19.7		7.00	0.057	0.006			8.71	
		N/Fe = 0.2, Fe_3O_4/Fe = 0.11	Fe_xN	72.40		7.00	0.027	0.003			4.16	
		N/Fe = 0.2, Fe_3O_4/Fe = 0.17	Fe_xN	68.70		7.00	0.072	0.007			11.09	
		N/Fe = 0.2, Fe_3O_4/Fe = 0.2	Fe_xN	65.30		7.00	0.043	0.004			6.67	
		N/Fe = 0.2, Fe_3O_4/Fe = 0.33	Fe_xN	55.50		7.00	0.061	0.006			9.40	
		N/Fe = 0.2, Fe_3O_4/Fe = 1	Fe_xN	16.80		7.00	0.065	0.006			9.98	
		N/Fe = 0.3, Fe_3O_4/Fe = 0.17	Fe_xN	70.60		7.00	0.172	0.017	0.005		26.53	
		N/Fe = 0.3, Fe_3O_4/Fe = 0.33	Fe_xN			7.00	0.081	0.008			12.51	
		N/Fe = 0.3, Fe_3O_4/Fe = 1	Fe_xN	16.70		7.00	0.063	0.006			9.71	
		N/Fe = 0.4, Fe_3O_4/Fe = 0.17	Fe_xN	74.20		7.00	0.052	0.005			8.01	
		N/Fe = 0.4, Fe_3O_4/Fe = 1	Fe_xN	22.90		7.00	0.050	0.005			7.70	
		N/Fe = 0.5, Fe_3O_4/Fe = 1	Fe_xN	21.90		7.00	0.056	0.006			8.69	

Primary Modification (Ligand)	Modification Method	Modification Dose	Fromed N species	Fe^0 content*	Target	pH initial	k_{obs} (1/hr)	k_M (L/h/g)	k_{SA} (L/h/m^2)	Electron Efficiency (%)	enhancement ratio (R) of k_M	Data Source
N-mZVI	Fe powder, Fe_3O_4, $NaNH_2$, Ball milled	N/Fe = 0.3, Fe_3O_4/Fe = 0.17	Fe_xN	70.60	*trans*-DCE	7.00	0.358	0.036			19.35	Gong/ Zhang [6]
		N/Fe = 0.3, Fe_3O_4/Fe = 0.17	Fe_xN	70.60	*cis*-DCE	7.00	0.307	0.031			16.20	
		N/Fe = 0.3, Fe_3O_4/Fe = 0.17	Fe_xN	70.60	1,1-DCE	7.00	0.187	0.019			24.70	
		N/Fe = 0.3, Fe_3O_4/Fe = 0.17	Fe_xN	70.60	VC	7.00	0.109	0.011			7.84	
		N/Fe = 0.3, Fe_3O_4/Fe = 0.17	Fe_xN	70.60	PCE	7.00	0.039	0.004			81.65	
nZVI	Gas-solid, nZVI			83.4	TCE	8.2	0.004	0.004	0.000			Brumo -vsky/ Filip[1]
						8.2	0.001	0.001				
N-nZVI	Gas-solid, nZVI, N_2/NH_3		γ′-Fe_xN	55.7	TCE	8.2	0.075	0.075	0.004		19.60	
			ε-Fe_xN	20.9		8.2	0.020	0.020	0.001		5.35	
			γ′-Fe_xN			8.2	0.028	0.028			28.00	
			ε-Fe_xN			8.2	0.001	0.001			10.20	
nZVI	Gas-solid, nZVI			83.4	PCE	8.2	0.004	0.004	0.000			Brumo -vsky/ Micic[10]
				83.4	*cis*-DCE	8.2	0.003	0.003	0.000			
S-nZVI	nZVI, Na_2S		FeS_x		PCE	8.2	0.003	0.003	0.000		0.61	
			FeS_x		*cis*-DCE	8.2	0.003	0.003	0.000		1.02	
N-nZVI	Gas-solid, nZVI, N_2/NH_3		γ′-Fe_xN	55.7	PCE	8.2	0.023	0.023	0.001		5.52	
			ε-Fe_xN	20.9	PCE	8.2	0.007	0.007	0.000		1.61	
			γ′-Fe_xN	55.7	*cis*-DCE	8.2	0.035	0.035	0.002		10.50	
			ε-Fe_xN	20.9	*cis*-DCE	8.2	0.028	0.028	0.001		8.39	
N-nZVI	Gas-solid, nZVI, N_2/NH_3		γ′-Fe_xN	57.4	TCE	5.80	0.029	0.058	0.002			Oborn-a/Filip[2]
			γ′-Fe_xN	57.4		5.70	0.048	0.096	0.004			
			γ′-Fe_xN	57.4		6.00	0.035	0.070	0.003			
			γ′-Fe_xN	57.4		6.30	0.036	0.072	0.003			
			γ′-Fe_xN	57.4		6.00	0.032	0.065	0.003			
			γ′-Fe_xN	57.4		6.20	0.036	0.071	0.003			
			γ′-Fe_xN	57.4		6.90	0.030	0.061	0.002			
			γ′-Fe_xN	57.4		6.10	0.028	0.056	0.002			
			γ′-Fe_xN	57.4		6.20	0.027	0.054	0.002			
			γ′-Fe_xN	57.4		5.80	0.024	0.048	0.002			
			γ′-Fe_xN	57.4		5.70	0.027	0.054	0.002			
			γ′-Fe_xN	57.4		6.20	0.028	0.055	0.002			
			γ′-Fe_xN	57.4		5.90	0.022	0.0448	0.0028			
			γ′-Fe_xN	57.4		7.80	0.036	0.071	0.003			
			γ′-Fe_xN	57.4		8.20	0.040	0.079	0.003			
			γ′-Fe_xN	57.4		9.20	0.065	0.129	0.005			
			γ′-Fe_xN	57.4		6.30	0.032	0.065	0.003			
			γ′-Fe_xN	57.4		6.40	0.002	0.004	0.000			
			γ′-Fe_xN	57.4		5.80	0.004	0.008	0.000			
			γ′-Fe_xN	57.4		6.70	0.017	0.034	0.001			
			γ′-Fe_xN	57.4		7.20	0.016	0.032	0.001			

Primary Modification (Ligand)	Modification Method	Modification Dose	Fromed N species	Fe^0 content*	Target	pH initial	k_{obs} (1/hr)	k_M (L/h/g)	k_{SA} (L/h/m^2)	Electron Efficiency (%)	enhancement ratio (R) of k_M	Data Source
N-nZVI	Gas-solid, nZVI, N_2/NH_3		γ'-Fe_xN	57.4		7.60	0.014	0.029	0.001			Oborn-a/Filip[2]
			γ'-Fe_xN	57.4		8.90	0.022	0.043	0.002			
			γ'-Fe_xN	57.4		7.50	0.007	0.015	0.001			
nZVI	$FeSO_4 \cdot 7H_2O$, $NaBH_4$				TCE		0.056	0.0183	0.0018			Meng/Xu[3]
N-nZVI	Solid-solid, gelatin, $C_4H_6O_4$, Fe	N/Fe= 0.01	Fe_4N	0.3	TCE							
		N/Fe= 0.02	Fe_4N							82.00		
		N/Fe= 0.05	Fe_4N									
		N/Fe= 0.07	Fe_4N				5.390	1.797	0.039	95.00	98	
		N/Fe= 0.13	Fe_4N	63			7.190	2.397	0.048		130	
N-nZVI	$C_8H_{12}ClNO_2$, $C_4H_{11}NO_3$, $FeCl_3$			26.9	TCE	6.50	0.021	0.021	0.000	85.50		Liang/Jiang[11]
				26.9		6.50	0.018	0.018	0.000	71.60		
				26.9			0.043	0.043	0.000			
				26.9			0.027	0.027	0.000			
mZVI	Fe powder, ball milled			94.9	TCE	7.00	0.005	0.001	0.001			Gong/He[5]
				94.9		8.00	0.017	0.002	0.003			
				94.9		9.00	0.000	0.000	0.000			
S-mZVI	S^0, Fe powder, Ball milled	S/Fe = 0.025	FeS_x		TCE	7.00	0.076	0.008			15.20	
		S/Fe = 0.025	FeS_x			8.00	0.041	0.004			2.41	
		S/Fe = 0.025	FeS_x			9.00	0.034	0.003			106.00	
		S/Fe = 0.025	FeS_x			10.00	0.031	0.003				
		S/Fe = 0.025	FeS_x			7.00	0.031	0.003			34.40	
N-mZVI	Fe powder, CH_4N_2O, Ball milled	N/Fe = 0.01	Fe-N_x	94.29	TCE	7.00	0.011	0.001	0.000		48.00	
		N/Fe = 0.02	Fe-N_x	92.33		7.00	0.240	0.024	0.014		9.00	
		N/Fe = 0.05	Fe-N_x	88.47		7.00	0.045	0.005	0.002	1.69	2.60	
		N/Fe = 0.1	Fe-N_x	93.44		7.00	0.013	0.001	0.001		2.80	
		N/Fe = 0.2	Fe-N_x	87.91		7.00	0.014	0.001	0.002		2.80	
		N/Fe = 0.05	Fe-N_x	88.47		8.00	0.021	0.002	0.001		1.24	
		N/Fe = 0.05	Fe-N_x	88.47		9.00	0.016	0.002	0.001		50.00	
		N/Fe = 0.05	Fe-N_x	88.47		10.00	0.012	0.001	0.000		7.65	
		N/Fe = 0.05	Fe-N_x	88.47		9.00	0.008	0.001	0.000		1.33	
S-N-mZVI	Fe powder, CH_4N_2S, Ball milled	N/Fe = 0.01, S/Fe = 0.005	Fe-N_x, FeS_x	91.95	TCE	7.00	0.056	0.006	0.002		11.20	
		N/Fe = 0.02, S/Fe = 0.01	Fe-N_x, FeS_x	90.77		7.00	0.290	0.029	0.011		58.00	
		N/Fe = 0.05, S/Fe = 0.025	Fe-N_x, FeS_x	88.00		7.00	0.330	0.033	0.022	12.93	66.00	
		N/Fe = 0.1, S/Fe = 0.05	Fe-N_x, FeS_x	86.64		7.00	0.100	0.010	0.005		20.00	
		N/Fe = 0.2, S/Fe = 0.1	Fe-N_x, FeS_x	85.69		7.00	0.012	0.001	0.001		2.40	
	S^0, Fe powder, CH_4N_2O, Ball milled	N/Fe = 0.05, S/Fe = 0.025	Fe-N_x, FeS_x	87.10	TCE	7.00	0.160	0.016	0.007		32.00	
			Fe-N_x, FeS_x	87.10		8.00	0.160	0.016	0.007		32.00	
			Fe-N_x, FeS_x	87.10		9.00	0.130	0.013	0.006	7.44	7.65	
			Fe-N_x, FeS_x	87.10		10.00	0.110	0.011	0.005			
	CH_4N_2S, Fe powder, Ball milled	N/Fe = 0.05, S/Fe = 0.025	Fe-N_x, FeS_x	88.00	TCE	8.00	0.200	0.020	0.014		11.80	
			Fe-N_x, FeS_x	88.00		9.00	0.190	0.019	0.013			

Primary Modification (Ligand)	Modification Method	Modification Dose	Fromed N species	Fe^0 content*	Target	pH initial	k_{obs} (1/hr)	k_M (L/h/g)	k_{SA} (L/h/m^2)	Electron Efficiency (%)	enhancement ratio (R) of k_M	Data Source
S-N-mZVI	CH_4N_2S, Fe powder, Ball milled		$Fe-N_x$, FeS_x	88.00		10.00	0.230	0.023	0.016			Gong/He[5]
			$Fe-N_x$, FeS_x	88.00		9.00	0.170	0.017	0.012		531.00	
	S^0, Fe powder, CH_4N_2O, Ball milled	N/Fe = 0.05, S/Fe = 0.025	$Fe-N_x$, FeS_x	87.10	TCE	9.00	0.120	0.012	0.006		375.00	
mZVI	Fe powder, ball milled			96.05	CF	7.00	0.024	0.002	0.006			Gong/He[12]
S-mZVI	S^0, Fe powder, Ball milled	S/Fe = 0.005	FeS_x	95.05	CF	7.00	0.110	0.011	0.017		4.58	
		S/Fe = 0.01	FeS_x	93.70		7.00	0.050	0.005	0.007		2.08	
		S/Fe = 0.025	FeS_x	90.56		7.00	0.031	0.003	0.003		1.29	
		S/Fe = 0.05	FeS_x	86.26		7.00	0.033	0.003	0.003		1.38	
N-mZVI	Fe powder, $C_3H_6N_6$, Ball milled	N/Fe = 0.01	$Fe-N_x$	93.10	CF	7.00	0.018	0.002	0.002		0.75	
		N/Fe = 0.025	$Fe-N_x$	90.95		7.00	0.042	0.004	0.005		1.75	
		N/Fe = 0.05	$Fe-N_x$	91.23		7.00	0.054	0.005	0.002		2.25	
		N/Fe = 0.1	$Fe-N_x$	90.93		7.00	0.023	0.002	0.001		0.96	
S-N-mZVI	S^0, Fe powder, $C_3H_6N_6$, Ball milled	N/Fe = 0.01, S/Fe = 0.005	$Fe-N_x$, FeS_x	91.16	CF	7.00	0.170	0.017	0.027		7.08	
		N/Fe = 0.01, S/Fe = 0.01	$Fe-N_x$, FeS_x	90.07		7.00	0.350	0.035	0.033		14.60	
		N/Fe = 0.01, S/Fe = 0.025	$Fe-N_x$, FeS_x	87.79		7.00	1.080	0.108	0.046		45.00	
		N/Fe = 0.01, S/Fe = 0.05	$Fe-N_x$, FeS_x	82.80		7.00	0.046	0.005	0.004		1.92	
		N/Fe = 0.025, S/Fe = 0.005	$Fe-N_x$, FeS_x	90.09		7.00	0.340	0.034	0.039		14.20	
		N/Fe = 0.025, S/Fe = 0.01	$Fe-N_x$, FeS_x	89.44		7.00	0.890	0.089	0.082		37.10	
		N/Fe = 0.025, S/Fe = 0.025	$Fe-N_x$, FeS_x	86.75		7.00	0.350	0.035	0.011		14.60	
		N/Fe = 0.025, S/Fe = 0.05	$Fe-N_x$, FeS_x	81.65		7.00	0.042	0.004	0.002		1.75	
		N/Fe = 0.05, S/Fe = 0.005	$Fe-N_x$, FeS_x	90.07		7.00	0.050	0.005	0.003		2.08	
		N/Fe = 0.05, S/Fe = 0.01	$Fe-N_x$, FeS_x	89.35		7.00	0.076	0.008	0.004		3.17	
		N/Fe = 0.05, S/Fe = 0.025	$Fe-N_x$, FeS_x	86.15		7.00	0.120	0.012	0.003		5.00	
		N/Fe = 0.05, S/Fe = 0.05	$Fe-N_x$, FeS_x	77.10		7.00	0.096	0.010	0.004		4.00	
		N/Fe = 0.1, S/Fe = 0.005	$Fe-N_x$, FeS_x	89.47		7.00	0.038	0.004	0.002		1.58	
		N/Fe = 0.1, S/Fe = 0.01	$Fe-N_x$, FeS_x	86.89		7.00	0.062	0.006	0.003		2.58	
		N/Fe = 0.1, S/Fe = 0.025	$Fe-N_x$, FeS_x	85.87		7.00	0.060	0.006	0.002		2.50	
		N/Fe = 0.1, S/Fe = 0.05	$Fe-N_x$, FeS_x	78.89		7.00	0.240	0.024	0.008		10.00	
mZVI	None				TCE	7.00	0.005	0.001	0.001			Gong/He[4]
mZVI	Fe powder, ball milled			94.56	TCE	7.00	0.017	0.002	0.001			
				94.56		4.00	0.009	0.001	0.000			
				94.56		6.00	0.005	0.001	0.000			
				94.56		7.00	0.002	0.000	0.000			
				94.56		8.00	0.000	0.000	0.000			
N-mZVI	Fe powder, $C_3H_6N_6$, Ball milled	N/Fe = 0.01	$Fe-N_x$	92.18	TCE	9.00	0.003	0.000	0.001		0.50	
		N/Fe = 0.025	$Fe-N_x$	95.66		7.00	0.005	0.000	0.001		0.92	
		N/Fe = 0.05	$Fe-N_x$	93.47		7.00	0.017	0.002	0.001		3.42	
		N/Fe = 0.1	$Fe-N_x$	94.89		7.00	0.013	0.001	0.001		2.67	
		N/Fe = 0.2	$Fe-N_x$	95.94		7.00	0.007	0.001	0.004		1.33	
		N/Fe = 0.3	$Fe-N_x$	90.58		7.00	0.005	0.001	0.004		1.08	

Primary Modification (Ligand)	Modification Method	Modification Dose	Fromed N species	Fe^0 content*	Target	pH initial	k_{obs} (1/hr)	k_M (L/h/g)	k_{SA} (L/h/m^2)	Electron Efficiency (%)	enhancement ratio (R) of k_M	Data Source
N-mZVI	Fe powder, $C_3H_6N_6$, Ball milled	N/Fe = 0.05	Fe-N_x	93.47		7.00	0.047	0.005	0.002		2.83	Gong/ He[4]
		N/Fe = 0.05	Fe-N_x	93.47		4.00	0.020	0.002	0.001		2.24	
		N/Fe = 0.05	Fe-N_x	93.47		6.00	0.017	0.002	0.001		3.33	
		N/Fe = 0.05	Fe-N_x	93.47		7.00	0.017	0.002	0.001		10.00	
		N/Fe = 0.05	Fe-N_x	93.47		8.00	0.040	0.004	0.002		96.00	
		N/Fe = 0.05	Fe-N_x	93.47		9.00	0.046	0.005	0.002			
		N/Fe = 0.05	Fe-N_x	93.47		10.00	0.032	0.003	0.002			
mZVI	Fe powder, ball milled			94.56	TCE	7.00	0.005	0.001	0.001	0.42		Gong/ He[13]
S-mZVI	S^0, Fe powder, Ball milled	S/Fe = 0.1	FeS_x	85.50	TCE	7.00	0.141	0.014	0.009	9.06	26.20	
N-mZVI	Fe powder, $C_3H_6N_6$, Ball milled	N/Fe = 0.05	Fe-N_x	93.47	TCE	7.00	0.017	0.002	0.001	0.33	3.06	
S-N-mZVI	S^0, Fe powder, $C_3H_6N_6$, Ball milled	N/Fe = 0.05, S/Fe = 0.01	Fe-N_x, FeS_x	92.15	TCE	7.00	0.455	0.046	0.016	3.34	84.40	
		N/Fe = 0.05, S/Fe = 0.02	Fe-N_x, FeS_x	90.25		7.00	0.473	0.047	0.014	4.60	87.80	
		N/Fe = 0.05, S/Fe = 0.05	Fe-N_x, FeS_x	86.87		7.00	0.520	0.052	0.013	7.22	96.50	
		N/Fe = 0.05, S/Fe = 0.1	Fe-N_x, FeS_x	79.52		7.00	0.841	0.084	0.030	11.63	156.00	
		N/Fe = 0.05, S/Fe = 0.2	Fe-N_x, FeS_x	72.20		7.00	0.275	0.028	0.011	47.45	51.00	
nZVI	Shrimp shell biochar, $FeSO_4 \cdot 7H_2O$, $NaBH_4$				TCEP	7.00	0.018	0.036	0.000			Yang/ Zhan[14]
S-nZVI	Shrimp shell biochar, $FeSO_4 \cdot 7H_2O$, $NaBH_4$,$Na_2S_2O_4$	S/Fe = 0.1	FeS_x		TCEP	7.00	0.231	0.462	0.002		12.83	
		S/Fe = 0.3	FeS_x			7.00	0.099	0.198	0.001		5.5	
		S/Fe = 0.05	FeS_x			7.00	0.065	0.130	0.001		3.61	
		S/Fe = 0.2	FeS_x			7.00	0.130	0.260	0.001		7.22	
		S/Fe = 0.1	FeS_x		CAP	7.00	1.020	2.040	0.008			
			FeS_x		NB	7.00	0.352	0.704	0.003			
			FeS_x		TBBPA	7.00	0.243	0.486	0.002			
S-mZVI	S^0, Fe powder, Ball milled	S/Fe = 0.32	FeS_x	72.30	TCE	7.00	0.130	0.013		10.20		Yu/ He[15]
			FeS_x	72.30		7.00	0.240	0.024		8.40		
			FeS_x	72.30		7.00	0.550	0.055		9.50		
S-nZVI-CMC	one-pot, CMC, $FeSO_4 \cdot 7H_2O$, $NaBH_4$, Na_2S for 30min	S/Fe = 0.05, CMC:0.3 wt%	FeS_x		TCE		0.110	0.110	0.006	1.20		Xu/He[16]
	two-pot, CMC, $FeSO_4 \cdot 7H_2O$, $NaBH_4$, Na_2S for 30min	S/Fe = 0.05, CMC:0.3 wt %	FeS_x	90.00	TCE		0.349	0.349	0.010	3.50		
	two-pot, CMC, $FeSO_4 \cdot 7H_2O$, $NaBH_4$, $Na_2S_2O_4$ for 30min	S/Fe = 0.2, CMC:0.3 wt%	FeS_x	20.00	TCE		1.140	1.140	0.033	13.00		
		S/Fe = 0.5, CMC:0.3 wt%	FeS_x	10.00			0.250	0.250	0.007			
		S/Fe = 0.2, CMC:0.3 wt%	FeS_x	20.00			1.100	1.100	0.032			
	one-pot, CMC, $FeSO_4 \cdot 7H_2O$, $NaBH_4$, $Na_2S_2O_4$ for 30min	S/Fe = 0.05, CMC:0.3 wt%			TCE		0.120	0.120	0.003			
nZVI-CMC	CMC, $FeSO_4 \cdot 7H_2O$, $NaBH_4$ for 30min	CMC:0.3 wt%			TCE		0.016	0.016	0.000	0.03		
mZVI	Fe powder, Ball milled				PCE	7.00	0.002	0.000	0.000	1.03		Wu/He[17]

Primary Modification (Ligand)	Modification Method	Modification Dose	Fromed N species	Fe^0 content*	Target	pH initial	k_{obs} (1/hr)	k_M (L/h/g)	k_{SA} (L/h/m^2)	Electron Efficiency (%)	enhancement ratio (R) of k_M	Data Source
mZVI	Fe powder, Ball milled				TCE	7.00	0.006	0.001	0.001	0.85		
					1, 1-DCE	7.00	0.004	0.000	0.001	1.11		
					trans-DCE	7.00	0.037	0.004	0.006	1.78		
					cis-DCE	7.00	0.016	0.002	0.003	1.88		
					VC	7.00	0.061	0.006	0.010	0.84		
S-mZVI	S^0, Fe powder, Ball milled	S/Fe = 0.2	FeS_x		PCE	7.00	0.004	0.000	0.000	5.34	2.85	Wu/He[17]
		S/Fe = 0.1	FeS_x	85.50		7.00	0.007	0.001	0.001	3.49	4.36	
		S/Fe = 0.05	FeS_x			7.00	0.009	0.001	0.001	1.83	5.52	
		S/Fe = 0.02	FeS_x			7.00	0.010	0.001	0.001	1.69	6.56	
		S/Fe = 0.2	FeS_x		TCE	7.00	0.218	0.022	0.014	5.34	37.10	
		S/Fe = 0.1	FeS_x	85.50		7.00	0.162	0.016	0.014	3.49	27.60	
		S/Fe = 0.05	FeS_x			7.00	0.137	0.014	0.011	1.83	23.30	
		S/Fe = 0.02	FeS_x			7.00	0.103	0.010	0.009	1.69	17.50	
		S/Fe = 0.2	FeS_x		1,1-DCE	7.00	0.007	0.001	0.000	5.34	1.95	
		S/Fe = 0.1	FeS_x	85.50		7.00	0.010	0.001	0.001	3.49	2.62	
		S/Fe = 0.05	FeS_x			7.00	0.007	0.001	0.001	1.83	2.01	
		S/Fe = 0.02	FeS_x			7.00	0.013	0.001	0.001	1.69	3.57	
		S/Fe = 0.2	FeS_x		*trans*-DCE	7.00	0.038	0.004	0.002	5.34	1.04	
		S/Fe = 0.1	FeS_x	85.50		7.00	0.035	0.004	0.003	3.49	0.96	
		S/Fe = 0.05	FeS_x			7.00	0.040	0.004	0.003	1.83	1.08	
		S/Fe = 0.02	FeS_x			7.00	0.043	0.004	0.004	1.69	1.17	
		S/Fe = 0.2	FeS_x		*cis*-DCE	7.00	0.001	0.000	0.000	5.34	0.05	
		S/Fe = 0.1	FeS_x	85.50		7.00	0.001	0.000	0.000	3.49	0.09	
		S/Fe = 0.05	FeS_x			7.00	0.003	0.000	0.000	1.83	0.19	
		S/Fe = 0.02	FeS_x			7.00	0.005	0.000	0.000	1.69	0.28	
		S/Fe = 0.2	FeS_x		VC	7.00	0.000	0.000	0.000	5.34	0.01	
		S/Fe = 0.1	FeS_x	85.50		7.00	0.001	0.000	0.000	3.49	0.02	
		S/Fe = 0.05	FeS_x			7.00	0.002	0.000	0.000	1.83	0.04	
		S/Fe = 0.02	FeS_x			7.00	0.004	0.000	0.000	1.69	0.07	
S-nZVI	Fe^{3+}, $NaBH_4$, $Na_2S_2O_4$	S/Fe = 0.006	FeS_x		PCE	6.50	0.000	0.000	0.000			Mo/Zhu[18]
		S/Fe = 0.007	FeS_x			6.50	0.002	0.002	0.000	0.76		
		S/Fe = 0.008	FeS_x			6.50	0.001	0.001	0.000			
		S/Fe = 0.01	FeS_x			6.50	0.001	0.001	0.000			
		S/Fe = 0.011	FeS_x			6.50	0.001	0.001	0.000	1.67		
		S/Fe = 0.015	FeS_x			6.50	0.000	0.000	0.000			
		S/Fe = 0.02	FeS_x			6.50	0.001	0.001	0.000	1.79		
		S/Fe = 0.038	FeS_x			6.50	0.002	0.002	0.000	2.33		
		S/Fe = 0.006	FeS_x		TCE	6.50	0.005	0.005	0.001			
		S/Fe = 0.007	FeS_x			6.50	0.074	0.074	0.010	4.31		
		S/Fe = 0.008	FeS_x			6.50	0.043	0.043	0.005			
		S/Fe = 0.01	FeS_x			6.50	0.064	0.064	0.006			
		S/Fe = 0.011	FeS_x			6.50	0.031	0.031	0.006	20.65		

Primary Modification (Ligand)	Modification Method	Modification Dose	Fromed N species	Fe^0 content*	Target	pH initial	k_{obs} (1/hr)	k_M (L/h/g)	k_{SA} (L/h/m^2)	Electron Efficiency (%)	enhancement ratio (R) of k_M	Data Source
		S/Fe = 0.015	FeS_x			6.50	0.021	0.021	0.005			
		S/Fe = 0.02	FeS_x			6.50	0.009	0.009	0.002	29.68		
		S/Fe = 0.038	FeS_x			6.50	0.014	0.014	0.002	24.86		
		S/Fe = 0.006	FeS_x			6.50	0.012	0.012	0.002			
		S/Fe = 0.007	FeS_x			6.50	0.050	0.050	0.007	1.39		
		S/Fe = 0.008	FeS_x			6.50	0.027	0.027	0.003			
		S/Fe = 0.011	FeS_x		*trans*-DCE	6.50	0.020	0.020	0.004	1.87		
		S/Fe = 0.015	FeS_x			6.50	0.012	0.012	0.003			
		S/Fe = 0.02	FeS_x			6.50	0.004	0.004	0.001	1.52		
		S/Fe = 0.038	FeS_x			6.50	0.008	0.008	0.001	1.68		
		S/Fe = 0.006	FeS_x			6.50	0.001	0.001	0.000			
		S/Fe = 0.007	FeS_x			6.50	0.007	0.007	0.001	1.20		
S-nZVI	Fe^{3+}, $NaBH_4$, $Na_2S_2O_4$	S/Fe = 0.008	FeS_x			6.50	0.004	0.004	0.000			Mo/ Zhu[18]
		S/Fe = 0.01	FeS_x		*cis*-DCE	6.50	0.001	0.001	0.000			
		S/Fe = 0.011	FeS_x			6.50	0.001	0.001	0.000	0.83		
		S/Fe = 0.015	FeS_x			6.50	0.001	0.001	0.000			
		S/Fe = 0.02	FeS_x			6.50	0.000	0.000	0.000	0.63		
		S/Fe = 0.038	FeS_x			6.50	0.001	0.001	0.000	0.51		
		S/Fe = 0.006	FeS_x			6.50	0.003	0.003	0.000			
		S/Fe = 0.007	FeS_x			6.50	0.008	0.008	0.001	4.07		
		S/Fe = 0.008	FeS_x			6.50	0.006	0.006	0.001			
		S/Fe = 0.01	FeS_x		VC	6.50	0.001	0.001	0.000			
		S/Fe = 0.011	FeS_x			6.50	0.000	0.000	0.000	12.89		
		S/Fe = 0.015	FeS_x			6.50	0.000	0.000	0.000			
		S/Fe = 0.02	FeS_x			6.50	0.000	0.000	0.000	8.93		
		S/Fe = 0.038	FeS_x			6.50	0.000	0.000	0.000	9.27		
nZVI	$FeSO_4{\cdot}7H_2O$, $NaBH_4$			93.10	TCE	8.00	0.000	0.000	0.000	2.40		
S-nZVI	$FeSO_4{\cdot}7H_2O$, $NaBH_4$, Na_2S	S/Fe = 0.2	FeS_x	86.60	TCE	8.00	0.010	0.039	0.002	72.00	277.00	He/He[19]
		S/Fe = 0.2	FeS_x	86.60		8.00	0.001	0.004	0.000		31.20	
nZVI	FeBH	0 g/L			TCE		0.075	0.037	0.001			
		S/Fe = 0.003	FeS_x				0.634	0.317	0.012			
		S/Fe = 0.015	FeS_x				0.935	0.468	0.015			
S-nZVI	$FeCl_3$, $NaBH_4$, $Na_2S_2O_4$	S/Fe = 0.03	FeS_x		TCE		1.060	0.530	0.016			Kim/ Chang[20]
		S/Fe = 0.06	FeS_x				1.590	0.795	0.019			
		S/Fe = 0.15	FeS_x				1.300	0.650	0.019			
		S/Fe = 0.0125	FeS_x			7.00	0.026	0.013				
		S/Fe = 0.025	FeS_x			7.00	0.023	0.011				
		S/Fe = 0.05	FeS_x			7.00	0.041	0.020				
S-nZVI	$FeSO_4{\cdot}7H_2O$, $NaBH_4$, $Na_2S_2O_4$	S/Fe = 0.1	FeS_x		TCE	7.00	0.127	0.064				Garcia /O'Car -roll[21]
		S/Fe = 0.3	FeS_x			7.00	0.056	0.028				
		S/Fe = 0.5	FeS_x			7.00	0.060	0.030				
	$FeSO_4{\cdot}7H_2O$, $NaBH_4$,	S/Fe =0.0125	FeS_x		TCE	7.00	0.027	0.013				

Primary Modification (Ligand)	Modification Method	Modification Dose	Fromed N species	Fe^0 content*	Target	pH initial	k_{obs} (1/hr)	k_M (L/h/g)	k_{SA} (L/h/m^2)	Electron Efficiency (%)	enhancement ratio (R) of k_M	Data Source
S-nZVI	$Na_2S_2O_3$	S/Fe = 0.025	FeS_x			7.00	0.055	0.027				Garcia /O'Car -roll[21]
		S/Fe = 0.05	FeS_x			7.00	0.064	0.032				
		S/Fe = 0.1	FeS_x			7.00	0.089	0.044				
		S/Fe = 0.3	FeS_x			7.00	0.064	0.032				
		S/Fe = 0.5	FeS_x			7.00	0.052	0.026				
	$FeSO_4\cdot 7H_2O$, $NaBH_4$, Na_2S	S/Fe = 0.0125	FeS_x		TCE	7.00	0.028	0.014				
		S/Fe = 0.025	FeS_x			7.00	0.038	0.019				
		S/Fe = 0.05	FeS_x			7.00	0.078	0.039				
		S/Fe = 0.1	FeS_x			7.00	0.115	0.057				
		S/Fe = 0.3	FeS_x			7.00	0.091	0.046				
		S/Fe = 0.5	FeS_x			7.00	0.111	0.056				
	$FeSO_4\cdot 7H_2O$, $NaBH_4$, $Na_2S_2O_4$	S/Fe = 0.0125	FeS_x		TCE	7.00	0.004	0.002				
		S/Fe = 0.025	FeS_x			7.00	0.003	0.001				
		S/Fe = 0.05	FeS_x			7.00	0.003	0.002				
		S/Fe = 0.1	FeS_x			7.00	0.007	0.003				
		S/Fe = 0.3	FeS_x			7.00	0.027	0.013				
		S/Fe = 0.5	FeS_x			7.00	0.034	0.017				
	$FeSO_4\cdot 7H_2O$, $NaBH_4$, $Na_2S_2O_3$	S/Fe = 0.0125	FeS_x		TCE	7.00	0.004	0.002				
		S/Fe = 0.025	FeS_x			7.00	0.003	0.001				
		S/Fe = 0.05	FeS_x			7.00	0.012	0.006				
		S/Fe = 0.1	FeS_x			7.00	0.011	0.006				
		S/Fe = 0.3	FeS_x			7.00	0.024	0.012				
		S/Fe = 0.5	FeS_x			7.00	0.021	0.011				
	$FeSO_4\cdot 7H_2O$, $NaBH_4$, Na_2S	S/Fe = 0.0125	FeS_x		TCE	7.00	0.006	0.003				
		S/Fe = 0.025	FeS_x			7.00	0.009	0.004				
		S/Fe = 0.05	FeS_x			7.00	0.026	0.013				
		S/Fe = 0.1	FeS_x			7.00	0.053	0.026				
		S/Fe = 0.3	FeS_x			7.00	0.045	0.023				
		S/Fe = 0.5	FeS_x			7.00	0.093	0.047				
	$FeSO_4\cdot 7H_2O$, $NaBH_4$, $Na_2S_2O_4$	S/Fe = 0.0125	FeS_x		TCE	7.00	0.007	0.004				
		S/Fe = 0.025	FeS_x			7.00	0.010	0.005				
		S/Fe = 0.05	FeS_x			7.00	0.006	0.003				
		S/Fe = 0.1	FeS_x			7.00	0.006	0.003				
		S/Fe = 0.3	FeS_x			7.00	0.022	0.011				
		S/Fe = 0.5	FeS_x			7.00	0.031	0.015				
	$FeSO_4\cdot 7H_2O$, $NaBH_4$, $Na_2S_2O_3$	S/Fe = 0.0125	FeS_x		TCE	7.00	0.003	0.002				
		S/Fe = 0.025	FeS_x			7.00	0.005	0.002				
		S/Fe = 0.05	FeS_x			7.00	0.008	0.004				
		S/Fe = 0.1	FeS_x			7.00	0.010	0.005				
		S/Fe = 0.3	FeS_x			7.00	0.021	0.011				
		S/Fe = 0.5	FeS_x			7.00	0.013	0.007				
	$FeSO_4\cdot 7H_2O$, $NaBH_4$, Na_2S	S/Fe = 0.0125	FeS_x		TCE	7.00	0.006	0.003				

Primary Modification (Ligand)	Modification Method	Modification Dose	Fromed N species	Fe^0 content*	Target	pH initial	k_{obs} (1/hr)	k_M (L/h/g)	k_{SA} (L/h/m^2)	Electron Efficiency (%)	enhancement ratio (R) of k_M	Data Source
S-nZVI	$FeSO_4 \cdot 7H_2O$, $NaBH_4$, Na_2S	S/Fe = 0.025	FeS_x			7.00	0.008	0.004				Garcia /O'Car -roll[21]
		S/Fe = 0.05	FeS_x			7.00	0.030	0.015				
		S/Fe = 0.1	FeS_x			7.00	0.054	0.027				
		S/Fe = 0.3	FeS_x			7.00	0.028	0.014				
		S/Fe = 0.5	FeS_x			7.00	0.063	0.031				
S-Fe_3O_4	$FeCl_3 \cdot 6H_2O$, $FeCl_2 \cdot 4H_2O$, NaOH, $Na_2S_2O_4$	S/Fe = 0.3	FeS_x		TCE	7.00	0.003	0.001				
		S/Fe = 0.5	FeS_x			7.00	0.005	0.002				
	$FeCl_3 \cdot 6H_2O$, $FeCl_2 \cdot 4H_2O$, NaOH, $Na_2S_2O_3$	S/Fe = 0.0125	FeS_x		TCE	7.00	0.001	0.000				
		S/Fe = 0.025	FeS_x			7.00	0.005	0.003				
		S/Fe = 0.05	FeS_x			7.00	0.009	0.005				
		S/Fe = 0.1	FeS_x			7.00	0.010	0.005				
		S/Fe = 0.3	FeS_x			7.00	0.005	0.003				
		S/Fe = 0.5	FeS_x			7.00	0.016	0.008				
	$FeCl_3 \cdot 6H_2O$, $FeCl_2 \cdot 4H_2O$, NaOH, Na_2S	S/Fe = 0.0125	FeS_x		TCE	7.00	0.007	0.004				
		S/Fe = 0.025	FeS_x			7.00	0.014	0.007				
		S/Fe = 0.05	FeS_x			7.00	0.019	0.010				
		S/Fe = 0.1	FeS_x			7.00	0.105	0.053				
		S/Fe = 0.3	FeS_x			7.00	0.151	0.075				
		S/Fe = 0.5	FeS_x			7.00	0.316	0.158				
	$FeCl_3 \cdot 6H_2O$, $FeCl_2 \cdot 4H_2O$, NaOH, $Na_2S_2O_4$	S/Fe = 0.5	FeS_x		TCE	7.00	0.004	0.002				
	$FeCl_3 \cdot 6H_2O$, $FeCl_2 \cdot 4H_2O$, NaOH, $Na_2S_2O_3$	S/Fe = 0.0125	FeS_x		TCE	7.00	0.001	0.000				
		S/Fe = 0.025	FeS_x			7.00	0.002	0.001				
		S/Fe = 0.05	FeS_x			7.00	0.002	0.001				
		S/Fe = 0.1	FeS_x			7.00	0.002	0.001				
		S/Fe = 0.3	FeS_x			7.00	0.001	0.000				
		S/Fe = 0.5	FeS_x			7.00	0.002	0.001				
	$FeCl_3 \cdot 6H_2O$, $FeCl_2 \cdot 4H_2O$, NaOH, Na_2S	S/Fe = 0.0125	FeS_x		TCE	7.00	0.004	0.002				
		S/Fe = 0.025	FeS_x			7.00	0.002	0.001				
		S/Fe = 0.05	FeS_x			7.00	0.003	0.001				
		S/Fe = 0.3	FeS_x			7.00	0.002	0.001				
		S/Fe = 0.5	FeS_x			7.00	0.004	0.002				
	$FeCl_3 \cdot 6H_2O$, $FeCl_2 \cdot 4H_2O$, NaOH, $Na_2S_2O_4$	S/Fe = 0.3	FeS_x		TCE	7.00	0.001	0.001				
		S/Fe = 0.5	FeS_x			7.00	0.002	0.001				
	$FeCl_3 \cdot 6H_2O$, $FeCl_2 \cdot 4H_2O$, NaOH, $Na_2S_2O_3$	S/Fe = 0.05	FeS_x		TCE	7.00	0.001	0.001				
		S/Fe = 0.1	FeS_x			7.00	0.001	0.000				
		S/Fe = 0.3	FeS_x			7.00	0.001	0.001				
		S/Fe = 0.5	FeS_x			7.00	0.001	0.000				
	$FeCl_3 \cdot 6H_2O$, $FeCl_2 \cdot 4H_2O$, NaOH, Na_2S	S/Fe = 0.025	FeS_x		TCE	7.00	0.001	0.000				
		S/Fe = 0.3	FeS_x			7.00	0.001	0.000				
		S/Fe = 0.5	FeS_x			7.00	0.001	0.001				

Primary Modification (Ligand)	Modification Method	Modification Dose	Fromed N species	Fe^0 content*	Target	pH initial	k_{obs} (1/hr)	k_M (L/h/g)	k_{SA} (L/h/m^2)	Electron Efficiency (%)	enhancement ratio (R) of k_M	Data Source
$S\text{-}Fe_3O_4$	$FeCl_3{\cdot}6H_2O$, $FeCl_2{\cdot}4H_2O$, NaOH, Na_2S	S/Fe = 0.05	FeS_x			7.00	0.065	0.033	0.000			Garcia /O'Carroll[21]
		S/Fe = 0.1	FeS_x			7.00	0.056	0.028	0.000			
		S/Fe = 0.5	FeS_x			7.00	0.144	0.072	0.001			
		S/Fe = 0.75	FeS_x			7.00	0.073	0.037	0.000			
		S/Fe = 1	FeS_x			7.00	0.036	0.018	0.000			
S-nZVI	$FeSO_4{\cdot}7H_2O$, $NaBH_4$, Na_2S	S/Fe = 0.2	FeS_x	57.00	TCE		0.559	0.559	0.024	0.48		Gong/He[22]
	$FeSO_4{\cdot}7H_2O$, $NaBH_4$, $Na_2S_2O_4$	S/Fe = 0.2	FeS_x		TCE		0.032	0.032	0.002			
	$FeSO_4{\cdot}7H_2O$, $NaBH_4$, $Na_2S_2O_3$	S/Fe = 0.2	FeS_x	65.00	TCE		0.075	0.075	0.005			
S-nZVI-CMC	CMC, $FeSO_4{\cdot}7H_2O$, $NaBH_4$, Na_2S	S/Fe = 0.05, CMC: 3 g/L	FeS_x		TCE		0.165	0.660	0.028			
		S/Fe = 0.1, CMC: 3 g/L	FeS_x				0.200	0.800	0.034			
		S/Fe = 0.2, CMC: 3 g/L	FeS_x	64.00			0.212	0.848	0.036			
		S/Fe = 0.4, CMC: 3 g/L	FeS_x				0.233	0.932	0.039			
	CMC, $FeSO_4{\cdot}7H_2O$, $NaBH_4$, $Na_2S_2O_4$	S/Fe = 0.05, CMC: 3 g/L	FeS_x		TCE		0.141	0.564	0.077			
		S/Fe = 0.1, CMC: 3 g/L	FeS_x				0.052	0.206	0.028			
		S/Fe = 0.2, CMC: 3 g/ L	FeS_x				0.055	0.218	0.030			
		S/Fe = 0.4, CMC: 3 g/L	FeS_x				0.066	0.264	0.036			
	CMC, $FeSO_4{\cdot}7H_2O$, $NaBH_4$, $Na_2S_2O_3$	S/Fe = 0.05, CMC: 3 g/L	FeS_x		TCE		0.080	0.320	0.041			
		S/Fe = 0.1, CMC: 3 g/L	FeS_x				0.095	0.380	0.048			
		S/Fe = 0.2, CMC: 3 g/L	FeS_x	53.00			0.084	0.336	0.043			
		S/Fe = 0.4, CMC: 3 g/L	FeS_x				0.089	0.356	0.045			
nZVI	$FeSO_4{\cdot}7H_2O$, $NaBH_4$				TCE	8.00	0.002	0.002		0.15		Gong/He[23]
						8.00	0.001	0.001		0.33		
						8.00	0.001	0.001		0.42		
						8.00	0.001	0.001		0.41		
						8.00	0.001	0.001		0.56		
						4.00	0.003	0.003		0.39		
						6.00	0.005	0.005		0.07		
						10.00	0.001	0.001		0.07		
						8.00	0.000	0.000		0.84		
						8.00	0.001	0.001		1.19		
						8.00	0.001	0.001		1.38		
						8.00	0.001	0.001		1.50		
							0.001	0.001		0.07		
							0.004	0.004		1.81		
							0.002	0.002		1.04		
S-nZVI	$FeSO_4{\cdot}7H_2O$, $NaBH_4$, Na_2S	S/Fe = 0.01	FeS_x		TCE	8.00	0.009	0.009		2.36	4.48	
		S/Fe = 0.02	FeS_x			8.00	0.026	0.026		3.30	12.40	
		S/Fe = 0.033	FeS_x			8.00	0.051	0.051		4.32	24.30	
		S/Fe = 0.05	FeS_x			8.00	0.085	0.085		5.94	40.50	
		S/Fe = 0.1	FeS_x			8.00	0.088	0.088		14.84	41.90	
		S/Fe = 0.2	FeS_x			8.00	0.032	0.032		29.19	15.20	

Primary Modification (Ligand)	Modification Method	Modification Dose	Fromed N species	Fe^0 content*	Target	pH initial	k_{obs} (1/hr)	k_M (L/h/g)	k_{SA} (L/h/m^2)	Electron Efficiency (%)	enhancement ratio (R) of k_M	Data Source
S-nZVI		S/Fe = 0.2	FeS_x			8.00	0.030	0.030		29.25	37.50	Gong/ He[23]
		S/Fe = 0.2	FeS_x			8.00	0.022	0.022		44.87	20.00	
		S/Fe = 0.2	FeS_x			8.00	0.020	0.020		56.75	35.70	
		S/Fe = 0.2	FeS_x			8.00	0.011	0.011		65.04		
		S/Fe = 0.2	FeS_x			4.00	0.088	0.088		4.54	30.30	
		S/Fe = 0.2	FeS_x			6.00	0.110	0.110		2.36	22.40	
		S/Fe = 0.2	FeS_x			10.00	0.038	0.038		14.40	52.80	
		S/Fe = 0.2	FeS_x			8.00	0.031	0.031		37.08	31.00	
		S/Fe = 0.2	FeS_x			8.00	0.020	0.020		44.83	22.20	
		S/Fe = 0.2	FeS_x			8.00	0.035	0.035		56.98	35.00	
		S/Fe = 0.2	FeS_x			8.00	0.029	0.029		65.69	26.40	
		S/Fe = 0.2	FeS_x				0.014	0.014		31.71	11.70	
		S/Fe = 0.2	FeS_x				0.059	0.059		15.32	15.50	
		S/Fe = 0.2	FeS_x				0.032	0.032		27.69	15.20	
mZVI	Fe powder, ball milled			92.70	TCE	7.00	0.012	0.001	0.006			Gong/ He[24]
				92.70		7.00	0.013	0.001	0.006			
				92.70		7.00	0.014	0.001	0.007			
				92.70		7.00	0.013	0.001	0.006			
				92.70		7.00	0.005	0.001	0.002			
				92.70		7.00	0.005	0.001	0.003			
				92.70		7.00	0.009	0.001	0.004			
				92.70		7.00	0.009	0.001	0.004			
S-mZVI	S^0, Fe powder, Ball milled	S/Fe = 0.1	FeS_x	85.50	TCE	7.00	0.146	0.015				
			FeS_x	85.50		7.00	0.146	0.015				
			FeS_x	85.50		7.00	0.125	0.013				
			FeS_x	85.50		7.00	0.100	0.010				
			FeS_x	85.50		7.00	0.071	0.007				
mZVI	Fe powder, ball milled			92.70	TCE	7.00	0.014	0.001	0.007			Gong/ He[25]
				92.70		7.00	0.010	0.001	0.005			
				92.70		7.00	0.014	0.001	0.007	1.40		
				92.70		7.00	0.017	0.002	0.008	1.20		
				92.70		7.00	0.088	0.009	0.042	0.70		
				92.70		7.00	0.018	0.002	0.008	1.10		
				92.70		7.00	0.138	0.014	0.065	3.20		
				92.70		7.00	0.179	0.018	0.085	1.40		
				92.70		7.00	0.108	0.011	0.052	5.60		
				92.70		7.00	0.096	0.010	0.046	5.30		
				92.70		7.00	0.014	0.001	0.007	1.40		
				92.70		7.00	0.012	0.001	0.006	1.50		
				92.70		7.00	0.013	0.001	0.006	1.50		
S-mZVI	S^0, Fe powder, Ball milled	S/Fe = 0.1	FeS_x	85.50	TCE	7.00	0.121	0.012	0.008	5.30		
			FeS_x	85.50		7.00	0.121	0.012	0.008	5.30		

Primary Modification (Ligand)	Modification Method	Modification Dose	Fromed N species	Fe^0 content*	Target	pH initial	k_{obs} (1/hr)	k_M (L/h/g)	k_{SA} (L/h/m^2)	Electron Efficiency (%)	enhancement ratio (R) of k_M	Data Source
S-mZVI	S^0, Fe powder, Ball milled	S/Fe = 0.1	FeS_x	85.50		7.00	0.146	0.015	0.010	5.10		Gong/He[25]
			FeS_x	85.50		7.00	0.133	0.013	0.009	5.20		
			FeS_x	85.50		7.00	0.142	0.014	0.010	5.50		
			FeS_x	85.50		7.00	0.133	0.013	0.009	5.30		
mZVI	Fe powder, ball milled			92.70	TCE	7.00	0.014	0.001	0.007	1.50		
				92.70		7.00	0.014	0.001	0.007	1.50		
				92.70		7.00	0.011	0.001	0.005	1.50		
mZVI	Fe powder, ball milled			92.70	TCE	6.00	0.603	0.060	0.287	0.95		Gu/He[26]
				92.70		7.00	0.008	0.001	0.004			
				92.70		8.00	0.004	0.000	0.002			
				92.70		9.00	0.000	0.000	0.000	5.30		
				92.70		10.00	0.000	0.000	0.000			
S-mZVI	S^0, Fe powder, Ball milled	S/Fe = 0.1	FeS_x	85.50	TCE	6.00	0.247	0.025	0.017	3.20		
			FeS_x	85.50		7.00	0.135	0.014	0.009			
			FeS_x	85.50		8.00	0.050	0.005	0.004			
			FeS_x	85.50		9.00	0.048	0.005	0.003	22.50		
			FeS_x	85.50		10.00	0.031	0.003	0.002	19.00		
mZVI	Fe powder, ball milled			92.70	TCE	7.00	0.004	0.000	0.002	0.19		Gu/He[27]
				92.70		7.00	0.001	0.005	0.024	8.00		
S-mZVI	S^0, Fe powder, Ball milled	S/Fe = 0.1	FeS_x	85.50	TCE	7.00	0.140	0.014	0.010	9.80		
			FeS_x	85.50	TCE	7.00	0.013	0.065	0.045	87.00		
mZVI	$FeCl_2$, $MgCl_2$				TCE	6.50	0.001	0.000	0.002	11.50		Cai/He[28]
S-mZVI	S^0, $FeCl_2$, $MgCl_2$	S/Fe = 0.1	FeS_x	83.20	TCE	6.50	0.500	0.050	0.028	80.00	500.00	
	S^0, $FeCl_2$, $CaCl_2$	S/Fe = 0.1	FeS_x	83.20	TCE	6.50	0.420	0.042	0.025	84.90	420.00	
	S^0, $FeCl_2$, KCl	S/Fe = 0.1	FeS_x	83.20	TCE	6.50	0.240	0.024	0.025	88.90	240.00	
	S^0, $FeCl_2$, NaCl	S/Fe = 0.1	FeS_x	83.20	TCE	6.50	0.200	0.020	0.027	86.40	200.00	
	S^0, $FeCl_2$	S/Fe = 0.1	FeS_x	83.20	TCE	6.50	0.210	0.021	0.021	91.20	210.00	
	S^0, $FeCl_2$, H_2O		FeS_x	83.20		6.50	0.230	0.023	0.025	91.70	230.00	
S-mZVI	ZVI, S^0	S/Fe = 0.1	FeS_x	82.40	TCE		1.200	0.120	0.387	5.34	800.00	Cai/He[29]
	ZVI, NaS	S/Fe = 0.1	FeS_x		TCE		0.068	0.007		3.49	45.30	
	ZVI, NaS_2O_3	S/Fe = 0.1	FeS_x		TCE		0.088	0.009		1.83	58.70	
			FeS_x				0.077	0.008			51.30	
	ZVI, NaS_2O_4	S/Fe = 0.1	FeS_x		TCE		0.054	0.005		1.69	36.00	
			FeS_x				0.047	0.005			31.30	

*: Fe^0 contents from hydrogen evolution after HCl addition

References

1. Brumovsky, M.; Oborna, J.; Micic, V.; Malina, O.; Kaslik, J.; Tunega, D.; Kolos, M.; Hofmann, T.; Karlicky, F.; Filip, J., Iron nitride nanoparticles for enhanced reductive dechlorination of trichloroethylene. *Environ. Sci. Technol.* **2022,** *56* (7), 4425-4436.
2. Oborná, J. K.; Brumovský, M.; Micić, V.; Kašlík, J.; Filip, J., Impact of groundwater solutes on the fate and reactivity of nanoscale iron nitride particles. *J. Environ. Chem. Eng.* **2025,** *13* (2), 115431.
3. Meng, F. X.; Xu, J.; Dai, H. W.; Yu, Y. L.; Lin, D. H., Even incorporation of nitrogen into Fe^0 nanoparticles as crystalline Fe_4N for efficient and selective trichloroethylene degradation. *Environ. Sci. Technol.* **2022,** *56* (7), 4489-4497.
4. Gong, L.; Qiu, X. J.; Tratnyek, P. G.; Liu, C. S.; He, F., FeN_X(C)-coated microscale zero-valent iron for fast and stable trichloroethylene dechlorination in both acidic and basic pH conditions. *Environ. Sci. Technol.* **2021,** *55* (8), 5393-5402.
5. Gong, L.; Chen, J. T.; Shen, L.; Zhang, Z. Z.; Xia, C. Y.; Wu, F.; Yao, Y. C.; Liu, C. S.; Liang, L. Y.; He, F., Unveiling the mechanistic role of surface nitrogen and sulfur in boosting the dechlorination performance of zero-valent iron. *ACS EST Engg.* **2024,** *4* (9), 2284-2293.
6. Gong, L.; Chen, J. T.; Zhan, G. M.; Zu, J. N.; Li, H.; He, F.; Tratnyek, P. G.; Zhang, L. Z., Mechanochemical molten-salt-assisted surface nitridation promotes electron transfer dechlorination of zerovalent iron. *Environ. Sci. Technol.* **2025,** *59* (19), 9802-9811.
7. Tratnyek, P. G.; Gao, Y.; Gong, L.; Mertsching, M., Abiotic reduction kinetics (ARK) database. *Zenodo* **2025**.
8. Shi, Y.; Wang, D.; Gao, F.; Liu, L.; Liu, Q.; Wang, L.; Tang, J., Mechanochemical nitridation of micron zero-valent iron for enhanced dechlorination of trichloroethylene: Mechanistic insights into nitrogen sources and ball milling conditions. *Sep. Purif. Technol.* **2024,** *337*, 126381.
9. Shi, Y.; Wang, D.; Guo, J.; Gao, F.; Liu, L.; Tang, J., Enhanced reductive degradation of trichloroethylene by ball milled nitridation of bimetallic Ni-ZVI: Combination effect of electron transfer and catalytic hydrogenation. *J. Clean. Prod.* **2024,** *470*.
10. Brumovsky, M.; Micic, V.; Oborna, J.; Filip, J.; Hofmann, T.; Tunega, D., Iron nitride nanoparticles for rapid dechlorination of mixed chlorinated ethene contamination. *J. Hazard. Mater.* **2023,** *442*, 129988.
11. Liang, Z.; Jiang, C.; Li, Y.; Liu, Y.; Yu, J.; Zhang, T.; Alvarez, P. J. J.; Chen, W., Single-atom iron can steer atomic hydrogen toward selective reductive dechlorination: Implications for remediation of chlorinated solvents-impacted groundwater. *Environ. Sci. Technol.* **2024,** *58* (26), 11833-11842.
12. Gong, L.; Chen, J. T.; Hu, Y.; He, K.; Bylaska, E. J.; Tratnyek, P. G.; He, F., Degradation of chloroform by zerovalent iron: Effects of mechanochemical sulfidation and nitridation on the kinetics and mechanism. *Environ. Sci. Technol.* **2023,** *57* (26), 9811-9821.
13. Gong, L.; Qiu, X. J.; Cheng, D.; Hu, Y.; Zhang, Z. Z.; Yuan, Q. S.; Yang, D. Z.; Liu, C. S.; Liang, L. Y.; He, F., Coincorporation of N and S into zero-valent iron to enhance TCE dechlorination: Kinetics, electron efficiency, and dechlorination capacity. *Environ. Sci. Technol.* **2021,** *55* (23), 16088-16098.
14. Yang, C.; Li, L.; Quan, K.; Liu, C.; Yang, L.; Zhu, C.; Yan, L.; Yuan, H.; Zhan, S., Sulfidated nanoscale zero-valent iron reductive degradation hydrophilic halogenated organic pollutant with the synergy of isolated Ca-Nx sites in biochar. *Chem. Eng. J.* **2025,** *504*.
15. He, F.; Yu, Y.; Wan, W.; Liang, L., Enhanced dechlorination of trichloroethene by sulfidated microscale zero-valent iron under low-frequency AC electromagnetic field. *J. Hazard. Mater.* **2022,** *423* (Pt A), 127020.
16. Xu, W.; Li, Z.; Shi, S.; Qi, J.; Cai, S.; Yu, Y.; O'Carroll, D. M.; He, F., Carboxymethyl cellulose stabilized and sulfidated nanoscale zero-valent iron: Characterization and trichloroethene dechlorination. *Appl. Catal. B Environ.* **2020,** *262*, 118303.
17. Wu, S.; Cai, S.; Qin, F.; He, F.; Liu, T.; Yan, X.; Wang, Z., Reductive dechlorination of chlorinated ethenes by ball milled and mechanochemically sulfidated microscale zero valent iron: A comparative study. *J. Hazard. Mater.* **2023,** *446*, 130730.
18. Mo, Y.; Xu, J.; Zhu, L., Molecular structure and sulfur content affect reductive dechlorination of chlorinated ethenes by sulfidized nanoscale zerovalent iron. *Environ. Sci. Technol.* **2022,** *56* (9), 5808-5819.
19. He, F.; Li, Z.; Shi, S.; Xu, W.; Sheng, H.; Gu, Y.; Jiang, Y.; Xi, B., Dechlorination of excess trichloroethene by bimetallic and sulfidated nanoscale zero-valent iron. *Environ. Sci. Technol.* **2018,** *52* (15), 8627-8637.
20. Kim, E. J.; Kim, J. H.; Azad, A. M.; Chang, Y. S., Facile synthesis and characterization of Fe/FeS nanoparticles for environmental applications. *ACS Appl. Mater. Interfaces* **2011,** *3* (5), 1457-62.
21. Nunez Garcia, A.; Lee, M.; Ding, L.; Liang, X.; Wang, C.; He, F.; O'Carroll, D. M., Sulfidation of magnetite for superior dechlorination of trichloroethene. *Environ. Sci. Technol.* **2025,** *59* (6), 3172-3182.
22. Gong, L.; Ying, S.; Xia, C.; Pan, K.; He, F., Carboxymethyl cellulose stabilization induced changes in particle characteristics and dechlorination efficiency of sulfidated nanoscale zero-valent iron. *Chemosphere* **2024,** *355*, 141726.

23. Gong, L.; Zhang, Z.; Xia, C.; Zheng, J.; Gu, Y.; He, F., A quantitative study of the effects of particle' properties and environmental conditions on the electron efficiency of Pd and sulfidated nanoscale zero-valent irons. *Sci. Total Environ.* **2022,** *853*, 158469.
24. Gong, L.; Qi, J.; Lv, N.; Qiu, X.; Gu, Y.; Zhao, J.; He, F., Mechanistic role of nitrate anion in TCE dechlorination by ball milled ZVI and sulfidated ZVI: Experimental investigation and theoretical analysis. *J. Hazard. Mater.* **2021,** *403*, 123844.
25. Gong, L.; Lv, N.; Qi, J.; Qiu, X.; Gu, Y.; He, F., Effects of non-reducible dissolved solutes on reductive dechlorination of trichloroethylene by ball milled zero valent irons. *J. Hazard. Mater.* **2020,** *396*, 122620.
26. Gu, Y. W.; Wang, B. B.; He, F.; Bradley, M. J.; Tratnyek, P. G., Mechanochemically sulfidated microscale zero valent iron: Pathways, kinetics, mechanism, and efficiency of trichloroethylene dechlorination. *Environ. Sci. Technol.* **2017,** *51* (21), 12653-12662.
27. Gu, Y. W.; Gong, L.; Qi, J. L.; Cai, S. C.; Tu, W. X.; He, F., Sulfidation mitigates the passivation of zero valent iron at alkaline pHs: Experimental evidences and mechanism. *Water Res.* **2019,** *159*, 233-241.
28. Cai, S.; Cao, Z.; Yang, L.; Wang, H.; He, F.; Wang, Z.; Xing, B., Cations facilitate sulfidation of zero-valent iron by elemental sulfur: Mechanism and dechlorination application. *Water Res.* **2023,** *242*, 120262.
29. Cai, S. C.; Chen, B.; Qiu, X. J.; Li, J. M.; Tratnyek, P. G.; He, F., Sulfidation of zero-valent iron by direct reaction with elemental sulfur in water: Efficiencies, mechanism, and dechlorination of trichloroethylene. *Environ. Sci. Technol.* **2021,** *55* (1), 645-654.
